# Checklist of Lichens and Lichenicolous Fungi from Mainland Portugal

**DOI:** 10.3390/jof12080549

**Published:** 2026-07-23

**Authors:** Palmira Carvalho, María Eugenia López de Silanes, Graciela Paz-Bermúdez, Joana Marques, Rui Figueira

**Affiliations:** 1 Museu Nacional de História Natural e da Ciência, Universidade de Lisboa, Rua da Escola Politécnica 58, 1250-102 Lisboa, Portugal; 2 Departamento de Enxeñaría dos Recursos Naturais e Medio Ambiente, Escola de Enxeñaría Forestal, Universidade de Vigo, 36005 Pontevedra, Spain; esilanes@uvigo.gal (M.E.L.d.S.); graciela@uvigo.gal (G.P.-B.); 3 CIBIO—Centro de Investigação em Biodiversidade e Recursos Genéticos, InBIO Laboratório Associado, Campus de Vairão, Universidade do Porto, 4485-661 Vairão, Portugal; joanamarques@cibio.up.pt (J.M.); ruifigueira@isa.ulisboa.pt (R.F.); 4 BIOPOLIS Program in Genomics, Biodiversity and Land Planning, CIBIO—Centro de Investigação em Biodiversidade e Recursos Genéticos, Campus de Vairão, 4485-661 Vairão, Portugal; 5 CIBIO—Centro de Investigação em Biodiversidade e Recursos Genéticos, InBIO Laboratório Associado, Instituto Superior de Agronomia, Universidade de Lisboa, Tapada da Ajuda, 1349-017 Lisboa, Portugal

**Keywords:** biodiversity, Iberian Peninsula, lichenised fungi, nomenclature, taxonomy, Western Europe

## Abstract

Mainland Portugal has a lichenological tradition spanning more than three centuries, yet it still lacks a modern, critically revised checklist of its lichen biota. This gap is particularly relevant given the increasing role of species checklists as authoritative references for DNA metabarcoding applications and the expansion of large biodiversity datasets, including those derived from citizen science initiatives. Here we present the first comprehensive checklist of lichens and lichenicolous fungi recorded from Mainland Portugal, based on the critical assessment of 43,718 bibliographic records drawn from 381 publications issued between 1788 and 2021. A total of 2012 species and infraspecific taxa are documented from a universe of 4440 taxonomic names, including synonyms. Nomenclature was updated in accordance with the ITALIC 8.0 database. Species distributions are provided by province, following the scheme adopted in Portuguese botanical floras, with additional information on substrates and altitudinal ranges where available. With over 2000 taxa recorded, Mainland Portugal can be considered a lichen-rich territory relative to its land area, comparing favourably with other European checklists. We discuss the factors underlying this diversity, with particular emphasis on the biogeographical position of Portugal at the south-western edge of the European continent.

## 1. Introduction

Checklists, or species inventories, are fundamental tools in biodiversity research and conservation [1,2]. The growing application of DNA metabarcoding and the expansion of large biodiversity databases have increased the demand for authoritative reference datasets capable of verifying taxonomic assignments and enhancing their accuracy, thereby providing standardised and critically evaluated accounts of taxa within a region or taxonomic group. Lichens account for about one-fifth of all described fungal species [3], and comprehensive checklists for this group serve to document distribution patterns, validate nomenclature, and detect spatial or temporal changes associated with environmental or anthropogenic drivers [4,5]. Such inventories further support ecological, biogeographical, and conservation research by enabling regional comparisons, identifying rare or endemic taxa, and informing monitoring strategies [6,7,8]. At a global scale, these resources contribute to the standardisation and accessibility of taxonomic information via platforms such as the Global Biodiversity Information Facility (GBIF) and the Catalogue of Life [9,10].

Despite their importance, a comprehensive checklist of lichens for Mainland Portugal remains lacking. The Iberian Peninsula, encompassing Portugal and Spain, harbours approximately half of all vascular plant species recorded in Europe [11], reflecting its exceptional biodiversity. This richness is particularly noteworthy given the region’s distinctive biogeographical position at south-western margin of the European continent. The convergence of Atlantic influence, Mediterranean climate, and palaeotropical relict vegetation creates conditions favourable to lichen diversity. Mild temperatures, elevated coastal humidity, and ancient laurel-forest fragments provide microhabitats suitable for tropical and pantropical lineages largely absent elsewhere in Europe. These climatic and environmental factors similarly govern the distribution and diversity of Iberian lichens, with potential implications for their responses to climate change [12].

Mainland Portugal has yielded numerous European records for families with tropical affinities, including *Graphidaceae* Dumort. [13], *Pyrenulaceae* Rabenh. [14], and *Peltigeraceae* Dumort. [15]. Species such as *Graphis striatula* (Ach.) Spreng, *Pyrenula complanata* (Mont.) Trevis. and several *Peltula* spp. represent significant range extensions into Europe [14,16,17]. Furthermore, the Iberian Peninsula served as a Pleistocene glacial refugium, enabling thermophilous and tropical-affinity organisms to persist through climatic oscillations that led to their elimination elsewhere on the continent. Collectively, these factors position Mainland Portugal as a key region for investigating lichen diversity at the temperate–tropical interface. Nevertheless, despite a lichenological research tradition spanning more than three centuries, the Portuguese lichen flora remains without a comprehensive and critically revised synthesis.

Portuguese lichenology has its origins in the mid-seventeenth century, developing alongside broader botanical studies. Early references include Grisley’s Viridarium Lusitanicum in 1661, later re-edited by Vandelli [18]. Brotero’s Flora Lusitanica (1804) [19] expanded the known flora, raising to 74 recorded species, and Arnold’s Lichenes Lusitaniae (1868) [20] constituted the first work devoted exclusively to Portuguese lichens. From the late nineteenth century onwards, contributions by Welwitsch, Henriques, Newton, Nylander, Harmand, Pereira Coutinho, and Gonçalo Sampaio collectively brought the total number of recorded species to approximately 600 species by the late 1930s.

Modern Iberian lichenology developed significantly through the work of Carlos das Neves Tavares in the mid-twentieth century [21,22,23], who made substantial contributions to the taxonomy of Portuguese and Macaronesian lichens. His premature death in 1972, compounded by the absence of immediate successors, resulted in a period of decline in national research. Between the 1970s and mid-1990s, foreign specialists such as Maurice Jones [24,25], Aptroot [26] and van den Boom [14,27] made sporadic contributions, preventing stagnation, though progress remained fragmented. From the mid-1990s onwards, renewed collaboration between Portuguese and international researchers revitalised the field. A landmark contribution of this period was the publication by Llimona and Hladun [28] of the Checklist of Iberian Lichens and Lichenicolous Fungi, which was since served as an essential reference framework for subsequent taxonomic and biogeographical studies.

To address the lack of a comprehensive checklist for Mainland Portugal, we compiled and critically reviewed historical and contemporary records from the published literature spanning the period 1788–2021. The work included the update of nomenclature and a table of the occurrences identified, which are published through GBIF. We further analyse the temporal and spatial distribution of records, sampling bias and main substrate of occurrences. This study provides a new standardised reference for future taxonomic, ecological, biogeographical, and conservation research on the lichenology in Portugal and in the Mediterranean region.

## 2. Materials and Methods

### 2.1. Study Area

This study encompasses Mainland Portugal, which is part of the Iberian Peninsula, in the south-western extremity of continental Europe. The geographic area extent, in the Northern Hemisphere, is between latitudes 37° N and 42° N and longitudes 6° W and 10° W. The territory exhibits a diverse orography, characterised by mountainous landscapes north of the Tagus River, and predominantly flat terrains in the south. Climate is principally influenced by latitude, topography, and proximity to the Atlantic Ocean.

Mainland Portugal is characterised by a transitional climate between Atlantic and Mediterranean influences, resulting in high spatial variability in temperature and precipitation patterns [29]. Overall, the climate is Mediterranean, characterised by hot, dry summers and mild, wet winters. In the north-western region, Atlantic climatic conditions prevail. Climatic variables such as precipitation and temperature display pronounced north–south and coastal–interior gradients, largely controlled by atmospheric circulation and topography [29]. Northern and coastal Atlantic areas are characterised by a humid regime with elevated annual precipitation, while southern and interior regions are subject to a more arid and continental climate, with very hot summers and low annual rainfall. Together, these climatic contrasts define a pronounced environmental gradient that exerts a strong influence on vegetation distribution across the territory.

The north-western region (Minho) ranks among the wettest areas in Europe, with mean annual precipitation exceeding 3000 mm in certain locations, whereas extensive areas of the interior Alentejo receive, on average, fewer than 500 mm annually. This marked spatial heterogeneity is accompanied by strong interannual variability, particularly in southern regions, contributing to recurrent droughts and heightened vulnerability to extreme climatic events [29]. Mean annual temperature ranges from approximately 10 °C in northern mountainous regions to circa 18 °C in the Algarve.

In this study, we used ‘provinces’ as geographic regions to describe the distribution of species across the country. These former administrative regions were established in 1936 and, although no longer in official use, are retained here for floristic purposes on account of their historical precedent and their recognition as biogeographically meaningful natural regions.

### 2.2. Bibliographic Review and Data Collection

This study drew upon scientific papers, monographs, and relevant historical works published between 1788 and 2021 that contain lichen occurrence records for Mainland Portugal. A total of 381 bibliographic sources were identified (Table S1), from which 43,718 species occurrences records were critically evaluated and extracted. Inclusion criteria encompassed all publications reporting lichen taxa with taxonomic, ecological, or distribution information. Publications lacking taxonomic data or species-level identification were excluded. Whilst every effort was made ensure comprehensive coverage of the available literature, access to certain works could not be secured. Only lichenised and lichenicolous fungi were considered.

Initial data collection involved the manual extraction of information from the publications into a structured spreadsheet comprising the following fields: scientific name, collection date, collector, determiner, locality, province, latitude, longitude, habitat, substrate, remarks, type information and bibliographic reference. This preliminary data table was subsequently converted into a dataset conforming to the Darwin Core (DwC) data standard [30], organised into five tables: Taxon (core), Occurrences, Extended Measurements or Facts, Species Distribution and Resource Relationship. The correspondence between the initial table structure and the Darwin Core schema is presented in Table S2.

### 2.3. Taxonomic Name Curation

A taxon list was derived from the original occurrence records by removing duplicate names as extracted from the original publications (verbatim). Each name was individually assessed for its current status (accepted, synonym, invalid), and the outcome recorded in a new field, TaxonStatus. Nomenclature was updated primarily in accordance with the ITALIC 8.0 database [5]. In addition, cross-validation was performed using additional taxonomic sources listed in Table S3, particularly for names not covered in ITALIC or when alternative treatments were necessary. The normalised name was recorded in a new field ScientificName. Where a taxon was determined to be currently a synonym, the corresponding accepted name was recorded in a new field AcceptedNameUsage. In cases involving name changes (e.g., correction of typos, normalisation of authorship), the original name as published was retained in a new field VerbatimIdentification, within in the Occurrence table. Taxa for which it was not possible to determine their validity and application, as well as those for which no current name could be found, were flagged as ‘nomina inquirenda’ in a new field NomenclaturalStatus.

The original remarks field consolidated annotations from the source publications concerning identifications requiring reconfirmation. These were transferred to a new field IdentificationRemarks in the Occurrence table. Additional remarks flagging species occurrences reported from an area as unlikely were recorded as ‘Doubtful record’ in a new field OccurrenceRemarks, likewise added to the Occurrence table. A separated category, ‘Excluded taxa’, was used in this field to list species previously reported from Portugal but subsequently excluded following verification as erroneous identifications, based on examination and re/identification of vouchers specimens.

Certain occurrence records were determined not to correspond to genuine occurrences in Portugal or were found to represent misidentifications or erroneous publications. Such records were excluded from both the taxon list and the occurrence list but are retained for reference in Table S4. This table compiles nine taxa falling into the following categories: (i) misidentifications: taxa published under erroneous identifications that were subsequently revised. Cross-referencing with more recent bibliographic sources and herbarium specimen reviews enabled to the correction of specimens previously published under incorrect names. Such corrections were applied only where it could be established with certainty that the records referred to the same specimen; (ii) erroneous synonymisations: taxa published under incorrect names as a result of erroneous synonymisation by the authors of certain works, frequently arising from the citation of older references without revision of the original specimens.

### 2.4. Data Processing, Georeferencing and Analysis

Occurrence records were thoroughly verified for data quality and consistency using OpenRefine [31]. For records containing geographic coordinates, the coordinate reference system (CRS) of the original values was identified and coordinates were converted to decimal latitude and longitude, using the WGS84 CRS. The converted values were recorded in new fields DecimalLatitude and DecimalLongitude, alongside two additional fields, CoordinatePrecision and CoordinateUncertaintyInMeters, following DwC concepts. Remaining records containing locality information were georeferenced in accordance with the protocol described by Chapman and Wieczorek [32]. The georeferencing workflow involved grouping occurrences by province and locality name and description, prioritising localities represented by more than ten occurrences. An additional field, GeoreferenceSources, documents the source used to obtain coordinates for each locality.

Data contained in the remaining fields of the original table (e.g., collection date, collector, determiner, substrate) were likewise normalised in accordance with DwC standard. OpenRefine’s clustering and transformation functions were extensively used to ensure consistency, for example, personal names and substrate type. In a number of cases, records were identified as duplicates, on the basis of the herbarium specimen catalogue numbers, cited across different publications. Such records were retained in the Occurrence table and additionally recorded in the ResourceRelationship table as duplicate entries. With respect to substrate type, the following categories were applied as interpretations of the substrate information documented in the source publication: ‘artificial’, ‘corticolous’, ‘lichenicolous’, ‘lignicolous’, ‘muscicolous’, ‘saxicolous’, ‘terricolous’, ‘foliicolous’, ‘endolithic’. These values were recorded in the Habitat field within the Occurrence table and additionally entered into the ExtendedMeasurementOrFact table, as values of the property ‘measurementType’. A Distribution table was prepared to document the presence of species across the provinces of Mainland Portugal. The five tables resulting from data processing—Taxon, Occurrence, ExtendedMeasurementOrFact, ResourceRelationship and Distribution—were structured according with the Darwin Core standard [33], with the latter three implemented as DwC extensions [34]. The complete dataset was published as a checklist dataset through GBIF [35].

Data was analysed using Python scripts implemented in Jupyter notebooks. For the development of scripts, Visual Studio Code (ver. 1.110.1) was used, including extension GitHub Copilot Chat (ver. 0.41) which used AI models (Claude Haiku 4.5) to create or edit code. Notebooks are available at https://github.com/rpfigueira/lichen-checklist-pt (accessed on 16 June 2026).

## 3. Results

### 3.1. Taxonomic Coverage

This checklist documents 2012 accepted species and infraspecific taxa (1843 species, 20 subspecies, 102 varieties and 47 forms). The compilation further includes 2409 synonyms, 124 taxa requiring further investigation to determine their validity (nomina inquirenda) and 22 nomina nuda. Nine species have been excluded from Mainland Portugal and 15 species are treated as doubtful occurrences.

**Figure 1:**
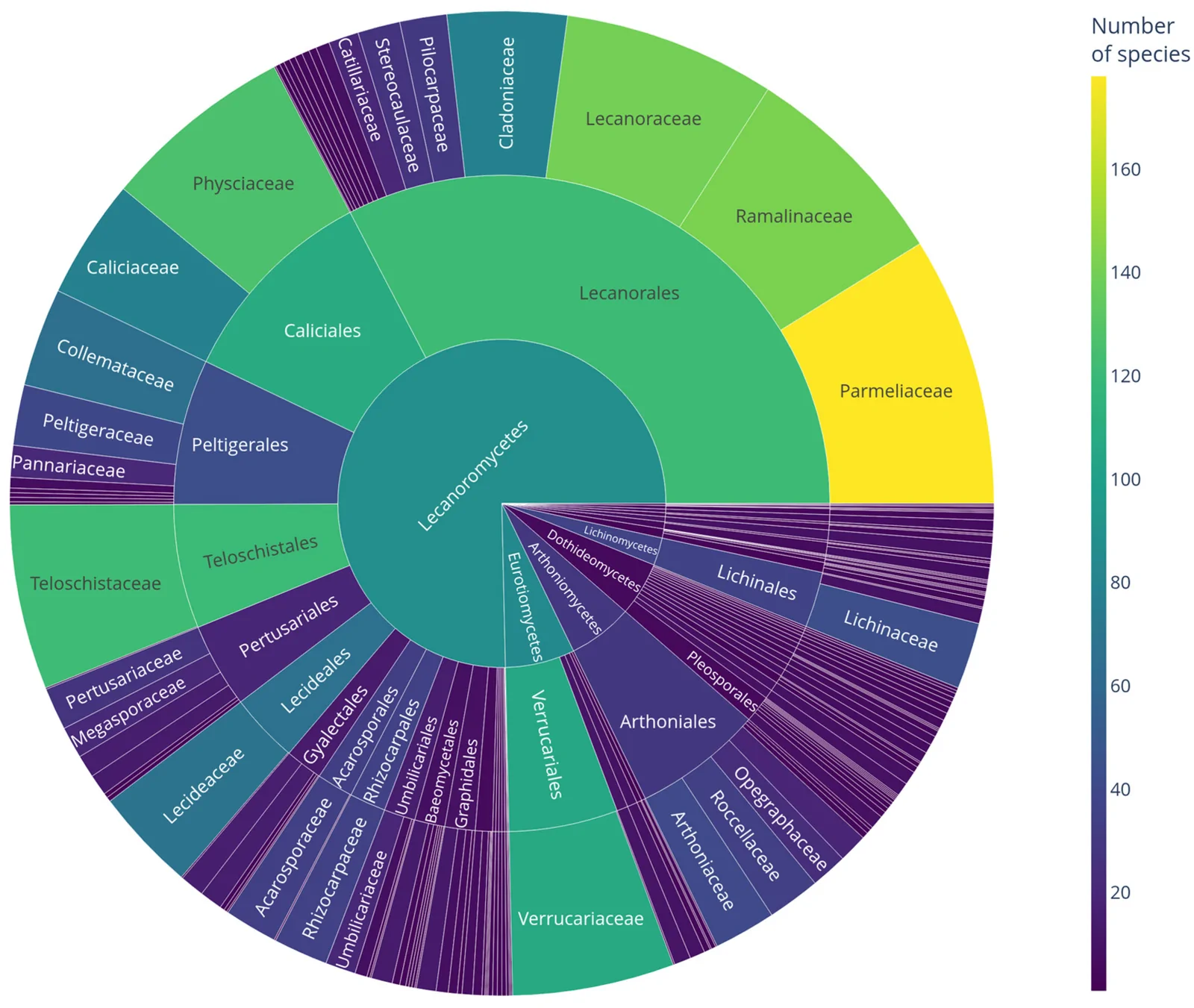
Taxonomic composition the species represented in the Portuguese checklist. The three taxonomic levels represented, from inner to outer rings, are class, order and family. The colour scale of the outer ring represents the number of species or infraspecific taxa belonging to that family. For clarity, labels corresponding to taxa with less than 20 counts are omitted, although all taxa are represented in the chart.

There are 11 fungal classes represented in the Mainland Portugal lichen flora. Most of the species documented belong to the class *Lecanoromycetes* O.E. Erikss. & Winka, which accounted for the majority of families and genera (Figure 1). Within this class, the order *Lecanorales* Nannf. is the most species-rich, with *Parmeliaceae* Bercht. & J. Presl, *Lecanoraceae* Körb., *Ramalinaceae* C. Agardh and *Cladoniaceae* Zenker representing the most diverse families. Other orders within *Lecanoromycetes* O.E. Erikss. & Winka, including *Caliciales* Bessey, *Teloschistales* D. Hawksw. & O.E. Erikss., *Peltigerales* Walt. Watson and *Pertusariales* M. Choisy ex D. Hawksw. & O.E. Erikss., contributed smaller but still substantial proportions of the total diversity. Additional classes, such as *Arthoniomycetes* O.E. Erikss. & Winka, *Eurotiomycetes* O.E. Erikss. & Winka, *Dothideomycetes* O.E. Erikss. & Winka and *Lichinomycetes* V. Reeb, Lutzoni & Cl. Roux, were present but comparatively less diverse. The taxa are distributed across 483 genera, 127 families and 56 orders (Table 1). The most represented genera, each comprising more than 60 species, are *Lecanora* Ach., *Cladonia* P. Browne and *Lecidea* Ach., while 219 genera are represented by a single species.

### 3.2. Distribution of Documented Occurrences

Analysis of the occurrence table compiled from published sources indicates that the most frequently recorded species are *Collema furfuraceum* (Schaer.) Du Rietz, *Flavoparmelia caperata* (L.) Hale, *Ramalina farinacea* (L.) Ach., *Lobaria pulmonaria* (L.) Hoffm. and *Evernia prunastri* (L.) Ach., each represented by more than 350 occurrences. *Collema furfuraceum* attained he highest number of occurrences by any single species, with 595 records. A total of 30 taxa are represented by 200 or more occurrences across the territory. Of the 2012 taxa documented, 388 are recorded only once (singletons).

**Figure 2:**
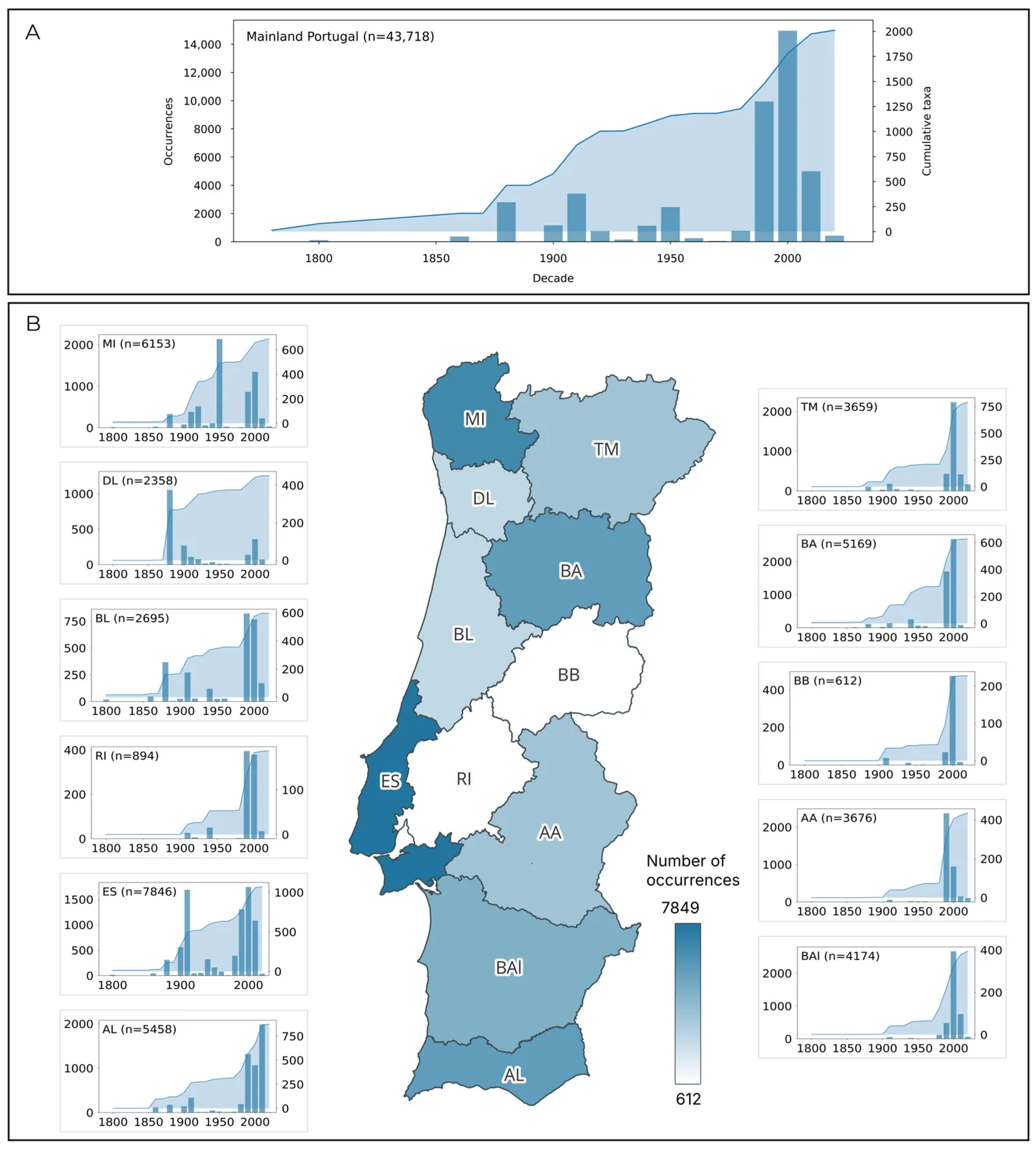
Temporal distribution of lichen occurrence records in Mainland Portugal (**A**) and across the eleven historical provinces (**B**) identified by codes. Bar charts represent occurrences by decades between 1800 and 2020, and the line chart behind shows the accumulated amount of taxa reported over time. The meaning of the axes for each chart in (**B**) are the same as in (**A**). The map illustrates the total number of occurrences in each province. Provinces are identified by the following codes: MI—Minho; TM—Trás-os-Montes e Alto Douro; DL—Douro Litoral; BA—Beira Alta; BL—Beira Litoral; BB—Beira Baixa; RI—Ribatejo; ES—Estremadura; AA—Alto Alentejo; BAl—Baixo Alentejo, AL—Algarve. The total numbers of occurrences are indicated by *n* next to the name or code of the region in each chart. Notice that axes are not standardised between provinces to improve readability of variations over time.

For flora studies, Mainland Portugal is typically geographically broken into provinces, a regional classification not matching any current administrative division in the country (see Figure 2). Of all taxa reported, only 66 taxa occur in all provinces, based on the published sources. The representativeness of taxa across the provinces in the country varies greatly, with the percentage of taxa in the province ranging between 9 and 53% (Table 2). The number of taxa is highly correlated with the number of occurrences (Pearson r = 0.86, *n* = 11), number of publications with records for that province (Pearson r = 0.93) and also number of singletons (Pearson r = 0.93). Estremadura yields the highest number of records (7860) and the greatest taxonomic richness (1077 taxa; 53%), corresponding to the highest number of publications. It is followed by Minho (6160 occurrences) and Algarve (5454 occurrences), although the latter is the province in which a single reference, the checklist of the Algarve [16], accounts for the highest percentage of all taxa known for the region (691 out of 872 taxa; 79.2%). Algarve presents greater taxonomic richness than Minho (872 and 690 taxa, respectively).

In contrast to the rest of the provinces, Ribatejo and Beira Baixa contributed the fewest records (894 and 612, respectively), with correspondingly low taxonomic coverage (9.1% and 11.2%), while Trás-os-Montes e Alto Douro, despite the moderate occurrence counts (3665) and a moderate number of references (105), ranks third in total number of taxa, yet shows a relatively high proportion of taxa represented by a single occurrence (252 singletons).

In general, there is no dominance of a single or few bibliographic references in informing the taxa present in a province, as the generally low values of the Simpson index based on the number of species reported by each reference seem to indicate. A broadly similar pattern is observed for lichenological richness, as expected.

Of the 2012 species recorded for Mainland Portugal, 577 taxa have been reported only once. Of these, 75% were published after the 1990s, coinciding with a renewed expansion of lichenological research in Portugal, whereas 20% date from the late nineteenth and early twentieth centuries. The latter predominantly comprise poorly known taxa that require taxonomic reassessment and formal validation. In addition, 29 of these taxa represent the only known occurrences in continental Europe, such as *Bacidina brittoniana* (Riddle) LaGreca & S. Ekman, *Caloplaca stanfordensis* H. Magn., *Canoparmelia aptata* (Kremp.) Elix & Hale, *Graphis crebra* Vain., *G. handelii* Zahlbr., *G. lineola* Ach and *G. plumierae* Vain. (Table S5).

### 3.3. Temporal Distribution of Documented Records

The temporal distribution of reported records across Mainland Portugal and its 11 provinces reflects the country’s history of scientific exploration from 1788 to the present (Figure 2). Temporal variation over time is considerable, indicating that the documentation of lichen diversity in Portugal has not followed a continuous or systematic approach but rather an opportunistic pattern characterised by discrete periods or activity punctuated by periods of minimal survey effort, a trend particularly pronounced in the interior regions of the country. Four distinct periods of higher numbers of occurrences reported can be identified, each corresponding to stepwise increments in the cumulative number of taxa reported. The first was observed in the 1880s, followed by 1920, 1950 and 1990–2010 (Figure 2A). At the provincial level, this general pattern exhibits considerable variation. Nonetheless, a consistent trend of stepwise increases in taxonomic reporting can be identified across provinces, with more recent decades generally corresponding to higher reporting rates. A notable exception is observed in Douro Litoral and Minho, where a marked decline in recorded occurrences is evident in recent decades, particularly in the former province.

This irregular temporal coverage is reflected in the cumulative number of species published over time. Pronounced increases are observed during two distinct periods, corresponding to the first and last decades of the 20th century. The former reflects the pioneering studies conducted by naturalists at the beginning of the century, while the latter reflects the high number of publications produced in recent decades, from 1995 onwards. Most provinces follow this broad two-phase pattern, albeit with notable differences in magnitude. Estremadura, Minho, Algarve and Trás-os-Montes e Alto Douro display the steepest cumulative curves, reflecting their consistently higher sampling effort, whereas Ribatejo and Beira Baixa show markedly flatter trajectories, with long intervals of negligible growth.

### 3.4. Areas of Higher Species Diversity

**Figure 3:**
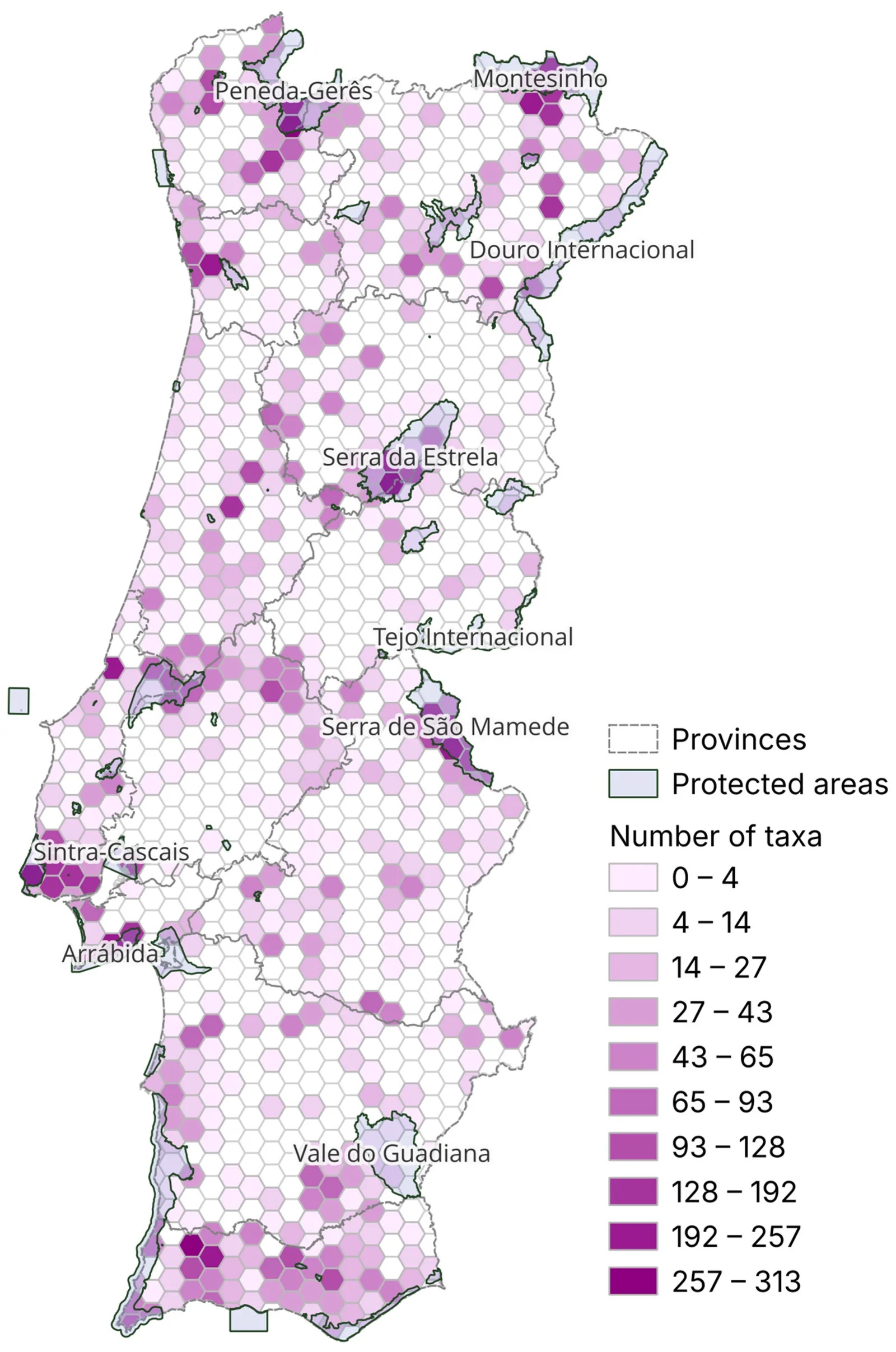
Species richness represented on a gridded map (10 km hexagon grid) for Mainland Portugal. On the map are depicted areas of higher numbers of species and the network of protected areas in the country. The protected areas with labels are mentioned in the text. The map is based on a total of 32,533 occurrences georeferenced in the country.

A detailed analysis of the species richness map, based on 32,533 georeferenced occurrences plotted on a 10 × 10 km grid, also reveals an uneven distribution of lichen diversity across Mainland Portugal (Figure 3). The first noteworthy observation is that almost half (49%) of the cells represented in the map have zero reported species. Among the grid cells with one or more species, several areas of particularly high species richness can be identified.

To identify those, we calculated the 95th percentile of the number of species per cell, which is 72. The total number of cells above that value is 58, which can be spatially clustered into 13 areas, seven of which are spatially associated with protected areas in the country. These include the Peneda-Gerês National Park in the north-west and Montesinho Natural Park in the north-east, both close to the border with Spain. The other protected areas are the Natural Parks of Serra da Estrela, Sintra-Cascais, Arrábida and Serra de São Mamede, which naturally represent the most intensively surveyed and species-rich areas in the country.

The grid cell with the highest number of species (313) is located in the Sintra Natural Park and represents the westernmost of continental Europe. However, not all protected areas are associated with high richness values. Most of the grid cells of three natural parks, namely Douro Internacional, Tejo Internacional and Vale do Guadiana, show low numbers of documented lichen species. Several other areas with high values can be related to mountainous areas or to locations which were targeted for specific purposes, like biomonitoring programmes or particular scientific projects.

### 3.5. Analysis of the Main Lichen Substrata

Of the total number of occurrences documented in the checklist, 78% (34,125 records) include information on the substrate, classified according to substrate classes. The most frequent substrate class is corticolous, accounting for 61% of total occurrences, followed by saxicolous (23%) and terricolous (7%). Lichenicolous species correspond to 1.7% of the occurrences, and residual percentages can be reported for lignicolous (0.37%) and foliicolous (0.03%) species. Interestingly, more than 600 records (1.6%) report occurrences on artificial substrate.

**Figure 4:**
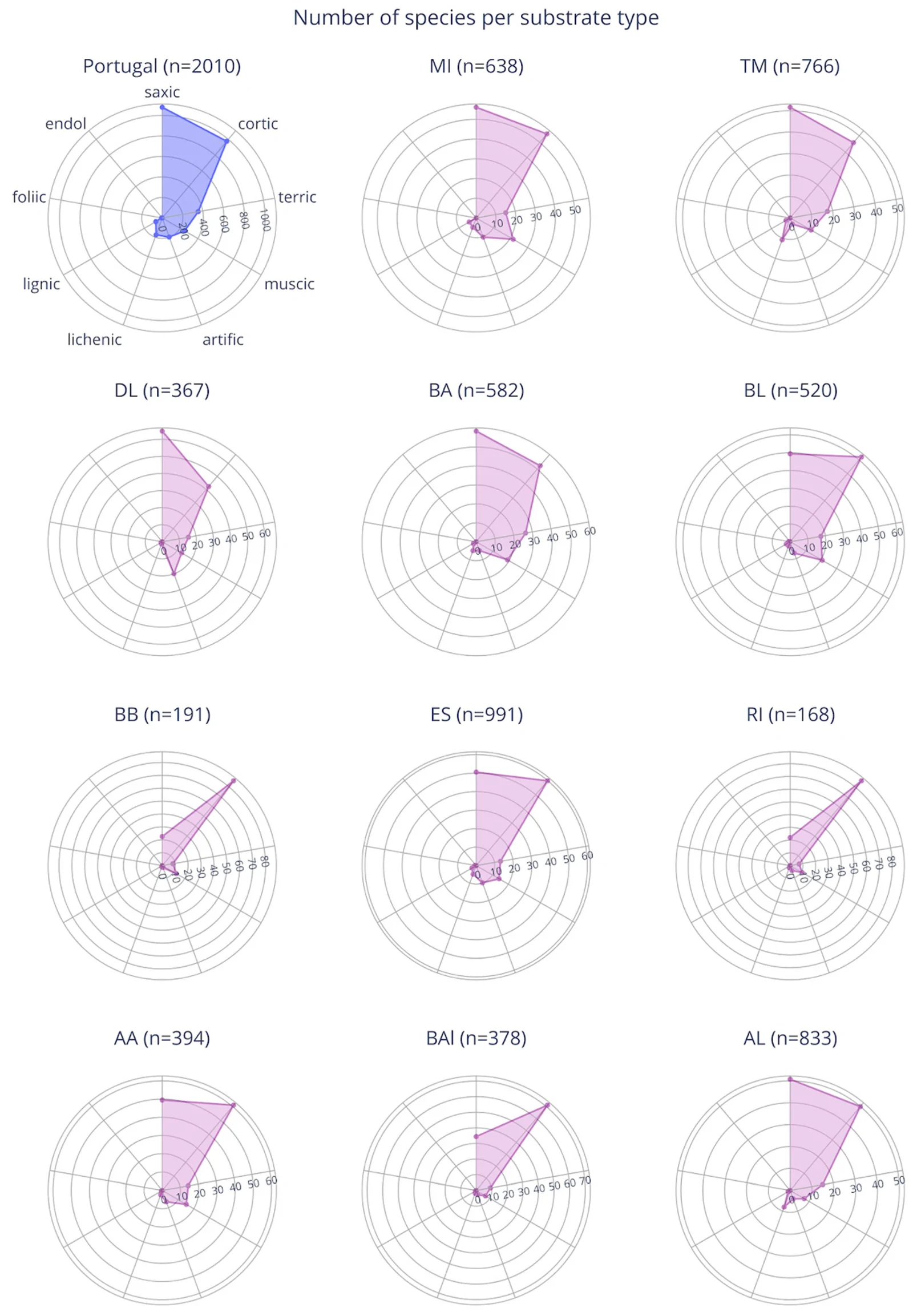
Total number of species per substrate type, for Mainland Portugal (top left plot in blue) and the eleven provinces. Provinces are identified by the following codes: MI—Minho; TM—Trás-os-Montes e Alto Douro; DL—Douro Litoral; BA—Beira Alta; BL—Beira Litoral; BB—Beira Baixa; RI—Ribatejo; ES—Estremadura; AA—Alto Alentejo; BAl—Baixo Alentejo; AL—Algarve. The title of each chart indicates the number of species for each region. Labels of each axis for the provinces’ charts follow the same order as in the Mainland Portugal chart, which correspond to: saxic—saxicolous; cortic—corticolous; terric—terricolous; muscic—muscicolous; artific—artificial; lichenic—lichenicolous; lignic—lignicolous; foliic—foliicolous; endol—endolithic.

Despite corticolous species accounting for approximately three times more citations than saxicolous species, the total number of saxicolous species reported for Portugal is slightly higher (Figure 4). This is a noteworthy finding, as corticolous taxa are generally considered the most abundant, having traditionally attracted greater interest within the lichenological community.

At the provincial level, saxicolous and corticolous taxa are the most representative groups. In Minho, Beira Alta, Douro Litoral, Trás-os-Montes e Alto Douro and Algarve, saxicolous species outnumber corticolous ones, in contrast to the remaining six provinces (Figure 4). The occurrence of muscicolous, terricolous and lichenicolous species, as well as those growing on artificial substrates, shows an uneven distribution across the different provinces. Lichenicolous fungi may be regarded as insufficiently studied in almost all provinces, with the exception of Trás-os-Montes e Alto Douro. It is also notable that foliicolous species are extremely scarce, with records from only two provinces: Estremadura and Beira Litoral.

## 4. Discussion

Although standard biogeographic classifications assign Mainland Portugal to only two regions—the majority of the country to the Mediterranean region and the north-western area to the Atlantic region—the country exhibits a combination of environmental gradients, substrate diversity and habitat heterogeneity that generates a broad range of ecological niches capable of sustaining high biodiversity. This is particularly relevant for fungi and lichens, which are highly sensitive to microclimatic conditions. The results of the present study demonstrate that Portugal harbours a rich lichen flora comprising more than 2000 species—a remarkable figure given the country’s total area of 92,000 km^2^. By comparison, Germany, which is approximately four times larger in area, records around 2600 taxa [36], while Italy, despite being three times the size of Portugal, accounts for approximately 2800 species [5,37].

This relatively high species richness recorded for Portugal may be attributable to its geographical position at the south-western edge of Europe, at the confluence of Atlantic, Mediterranean and continental climatic influences. This biogeographical setting, combined with complex orography and high habitat diversity, sustains a highly diverse lichenised fungal community. Notably, Switzerland, with approximately half the area of Portugal (~41,000 km^2^), records 2099 lichenised species and 424 lichenicolous fungi [38]. These comparisons highlight that geographic area alone is insufficient to explain patterns of species richness; additional drivers such as topographic heterogeneity, habitat diversity, the extent of natural and semi-natural habitats, woodland cover, and prevailing environmental conditions are likely to play a significant role [39]. Nevertheless, it is also possible that lichen biodiversity in Portugal remains underestimated, given the absence of systematic surveys across large portions of the territory.

The distribution of occurrence records in the dataset reveals considerable geographic sampling bias. Collection records are predominantly concentrated in a few well-studied areas associated with both historical and contemporary lichenological surveys. The most intensively sampled regions can generally be attributed to several main motivations underlying lichen surveys. First, many sampled localities are situated in the vicinity of museums and universities where lichenologists are based, with proximity being a primary determinant of sampling effort. Second, botanical surveys have traditionally focused on natural parks and protected areas, such as Serra da Estrela, the highest mountain in Mainland Portugal, or areas recognised for their high biodiversity value, such as Peneda-Gerês National Park. Accessibility and specific geographic or taxonomic interest have been documented as significant drivers of sampling bias [40]. In some instances, protected area management authorities have commissioned targeted surveys to obtain comprehensive species inventories [41]. Finally, a number of localities have been the focus of floristic or ecological studies conducted within the framework of biomonitoring programmes for environmental quality assessment, particularly in areas associated with industrial activity [42,43], power generation facilities [44,45], or mine sites [46,47]. In contrast, large portions of the territory, particularly inland regions, remain poorly characterised with respect to lichen biodiversity, whereas coastal and urban-adjacent areas have received disproportionately greater research attention.

The high proportion of singleton taxa recorded across almost all provinces further underscores the fragmentary nature of the current dataset and highlights the need for continued, systematic field surveys throughout the country. Singletons may result from insufficient sampling effort, taxonomic identification uncertainties, or genuinely rare taxa, with inadequate survey effort considered the principal explanation in most cases [48]. The fact that 29 of these taxa represent a single known occurrence in continental Europe further emphasises the urgency of expanding survey efforts and reassessing previously collected material.

The cumulative number of species recorded over time reveals variable rates of increase, with pronounced growth in two distinct periods corresponding to the first and last decades of the 20th century. The earlier period reflects the pioneering contributions of naturalists active at the turn of the century, while the more recent acceleration, from 1995 onwards, is associated with a marked increase in publications arising from the inventory studies and research projects previously mentioned, as well as the expansion of systematic surveys and the progressive digitisation of herbarium collections. Notably, the geographical coverage characterising these two periods of lichenological growth differs considerably. Whereas the more recent period encompasses collections spanning nearly all Portuguese provinces and a broad range of localities and habitat types, the early 20th-century records were largely confined to the provinces where active naturalists resided and worked, reflecting the logistical constraints on travel to inland regions that prevailed at the time.

Analysis of substrate preferences indicates that saxicolous species predominate in terms of taxon richness, despite corticolous species having approximately three times as many occurrence records. This pattern is consistent with findings from other checklists in which saxicolous species constitute the most frequent substrate type, as reported for Italy [37], the Alps [49], and Hong Kong [50]. In the Italian checklist, for instance, 55.8% of 2875 infrageneric taxa are classified as saxicolous and 33.1% epiphytic (corticolous).

For Mainland Portugal, we observed relatively similar values between the two types of lichen substrate, with 34.7% of taxa reported as saxicolous and 31.4% as corticolous. Nevertheless, corticolous lichens account for the largest proportion of occurrence records overall. This apparent discrepancy likely reflects the nature of lichenological research conducted in Portugal over the last three decades, which has largely focused on air-quality biomonitoring and biodiversity assessments in forest ecosystems and *montado* landscapes. These studies have primarily targeted epiphytic (corticolous) lichen communities, resulting in a greater number of occurrence records for corticolous taxa.

Saxicolous communities, by contrast, although supporting a higher total number of species, have been comparatively neglected, as they typically occur in remote or less accessible rocky outcrops and mountain ranges. In recent years, a series of studies has targeted saxicolous communities, particularly in Trás-os-Montes e Alto Douro and Douro Litoral, contributing to a significant increase in the number of recorded taxa [51,52]. However, this might not have been sufficient to ensure the sufficient inventory of saxicolous taxa in the country. The remarkable geological diversity of Mainland Portugal, encompassing granites, schists, limestones, and ultramafic rocks, provides a wide range of microhabitats that support high saxicolous species richness, which is nonetheless likely still underestimated due to insufficient sampling effort.

Lichenicolous fungi remain insufficiently studied, likely because these species are frequently overlooked due to their small size and the taxonomic expertise required for their identification [53], factors which, combined with the limited research attention they have historically received in the country, result in comparatively few reported occurrences.

The present checklist consolidates current knowledge on lichens and lichenicolous fungi in Mainland Portugal and is intended to serve not only as a comprehensive taxonomic reference but also as an infrastructural resource supporting further research in lichen biodiversity. Among its potential applications, the checklist may underpin the development of DNA barcode reference libraries and the identification of existing sampling and sequence gaps, an approach observed for other biological groups, including phytoplankton [54], aquatic biota [55], and mammals [56]. The strategic importance of such initiatives has been widely recognised, as the construction of robust DNA barcode reference libraries requires adherence to best practices in data management and field sampling, grounded in validated local checklists, and necessitates rigorous library curation including expert taxonomic validation [57]. Such reference frameworks are essential to support species discovery, biodiversity monitoring, and the improvement of taxonomic precision in DNA-metabarcoding-based identification pipelines.

Although the study was conducted following a systematic mobilisation of lichen occurrences from bibliographic sources in Mainland Portugal, and represents the most comprehensive account to date, we would caution users of the dataset regarding potential limitations of such data in terms of taxa distribution.

Firstly, only published sources were considered, and consequently occurrences or specimens from herbaria, or observations never cited in a scientific paper, are not included. Although it is unlikely that taxa represented in herbaria have not been captured from at least one published source, and thus the completeness of the checklist is expected to be high, the implications for chorological information may be considerable. While province-level occurrence data could be determined for 98% of records, geographic coordinates could be retrieved for only 75% of records. As a result, the distribution of taxa for higher resolution, for example, at the municipality level, may be incompletely represented.

Secondly, common species are frequently overlooked in the field, and their representation in herbaria does not match their abundance [58]. *Xanthoria parietina* (L.) Th. Fr. provides a pertinent example: despite being conspicuous, it is rarely reflected in collections owing to sampling bias, and in our dataset, it does not appear in the top 20 of most cited species. In contrast, *Lobaria pulmonaria* (L.) Hoffm. is the fourth most cited species, likely reflecting collector preference for rare or notable taxa rather than true relative abundance.

A systematic analysis of the current database to identify spatial and taxonomical gaps and bias represents the logical step in designing future field surveys that are systematic and geographically comprehensive. A more balanced sampling effort across underrepresented regions, together with the integration of additional herbarium data, will be essential to produce a more accurate and complete characterisation of lichen species richness and distribution across the territory.

## 5. Conclusions

In this study we report the occurrence of 2012 lichen and lichenicolous fungi taxa in Mainland Portugal, as documented from published sources. The distribution of the occurrences is considered to have significant spatial bias, leaving substantial areas of the country underrepresented. This checklist should be regarded as a baseline and a living document rather than a definitive account of lichen diversity in Portugal. Lichenological knowledge is inherently dynamic, and the inventory of lichen flora must be continuously updated as new collections, taxonomic revisions, and floristic studies become available. Priority should be given to undersampled regions that are potentially rich in biodiversity, particularly protected areas such as national parks and nature reserves, where habitat integrity and microclimate diversity are likely to support lichen communities yet to be documented. The integration of herbarium data, targeted floristic studies, and environmental biomonitoring programmes will be crucial to improve geographical coverage and to detect potential shifts in lichen distribution associated with ongoing climate and land-use change. Only through sustained and geographically comprehensive inventory efforts will it be possible to produce a truly representative picture of the lichen flora of Mainland Portugal.

## Figures and Tables

**Table 1:** Taxonomic summary of orders, families and genera recorded in the checklist of Mainland Portugal, with the corresponding number of species. Bracketed values indicate the number of subordinate taxa (families per order, genera per family).

Orders	Families	Genera	Number of Species
*Abrothallales* (1)	*Abrothallaceae* (1)	*Abrothallus*	9
*Acarosporales* (2)	*Acarosporaceae* (6)	*Acarospora*	24
		*Caeruleum*	1
		*Myriospora*	2
		*Pleopsidium*	3
		*Polysporina*	2
		*Sarcogyne*	3
	*Eigleraceae* (1)	*Eiglera*	1
*Agaricales* (1)	*Hygrophoraceae* (1)	*Lichenomphalia*	2
*Arctomiales* (1)	*Arctomiaceae* (1)	*Arctomia*	2
*Arthoniales* (7)	*Arthoniaceae* (8)	*Arthonia*	31
		*Arthothelium*	1
		*Briancoppinsia*	1
		*Coniocarpon*	2
		*Naevia*	3
		*Pachnolepia*	1
		*Snippocia*	1
		*Tylophoron*	1
	*Chrysotrichaceae* (1)	*Chrysothrix*	4
	*Incertae sedis* (2)	*Bactrospora*	1
		*Felipes*	1
	*Lecanographaceae* (5)	*Alyxoria*	8
		*Lecanographa*	5
		*Phacographa*	1
		*Plectocarpon*	2
		*Zwackhia*	3
	*Opegraphaceae* (3)	*Ingaderia*	3
		*Opegrapha*	20
		*Paralecanographa*	1
	*Roccellaceae* (14)	*Chiodecton*	3
		*Cresponea*	1
		*Dendrographa*	2
		*Dirina*	5
		*Diromma*	1
		*Enterographa*	5
		*Gyrographa*	2
		*Lecanactis*	1
		*Ocellomma*	1
		*Pseudoschismatomma*	1
		*Psoronactis*	1
		*Roccella*	7
		*Schismatomma*	4
		*Syncesia*	1
	*Roccellographaceae* (2)	*Fulvophyton*	1
		*Roccellographa*	2
*Asterinales* (1)	*Asterinaceae* (2)	*Labrocarpon*	1
		*Melaspileella*	1
*Atheliales* (1)	*Atheliaceae* (1)	*Athelia*	1
*Baeomycetales* (7)	*Arctomiaceae* (1)	*Gabura*	1
	*Arthrorhaphidaceae* (1)	*Arthrorhaphis*	2
	*Baeomycetaceae* (2)	*Ainoa*	1
		*Baeomyces*	1
	*Hymeneliaceae* (3)	*Hymenelia*	1
		*Ionaspis*	2
		*Tremolecia*	1
	*Protothelenellaceae* (2)	*Protothelenella*	1
		*Thrombium*	1
	*Trapeliaceae* (4)	*Placynthiella*	3
		*Rimularia*	1
		*Trapelia*	4
		*Trapeliopsis*	6
	*Xylographaceae* (2)	*Lambiella*	3
		*Xylographa*	2
*Caliciales* (2)	*Caliciaceae* (13)	*Acolium*	2
		*Amandinea*	8
		*Buellia*	37
		*Calicium*	13
		*Dimelaena*	2
		*Diploicia*	2
		*Diplotomma*	4
		*Endohyalina*	2
		*Orcularia*	1
		*Pyxine*	1
		*Sculptolumina*	1
		*Tetramelas*	3
		*Thelomma*	3
	*Physciaceae* (12)	*Anaptychia*	5
		*Coscinocladium*	1
		*Heterodermia*	3
		*Hyperphyscia*	1
		*Leucodermia*	1
		*Phaeophyscia*	11
		*Physcia*	24
		*Physciella*	1
		*Physconia*	11
		*Polyblastidium*	2
		*Rinodina*	66
		*Tornabea*	1
*Candelariales* (1)	*Candelariaceae* (2)	*Candelaria*	3
		*Candelariella*	7
*Capnodiales* (1)	*Mycosphaerellaceae* (2)	*Sphaerellothecium*	1
		*Stigmidium*	6
*Chaetothyriales* (2)	*Herpotrichiellaceae* (2)	*Capronia*	1
		*Cladophialophora*	2
	*Incertae sedis* (1)	*Lichenodiplis*	1
*Collemopsidiales* (1)	*Xanthopyreniaceae* (2)	*Collemopsidium*	4
		*Didymellopsis*	2
*Coniocybales* (1)	*Coniocybaceae* (1)	*Chaenotheca*	7
*Corticiales* (1)	*Corticiaceae* (1)	*Marchandiomyces*	1
*Diaporthales* (1)	*Gnomoniaceae* (1)	*Phoma*	2
*Dothideales* (1)	*Incertae sedis* (4)	*Cercidospora*	1
		*Endococcus*	2
		*Homostegia*	1
		*Monodictys*	2
*Eremithallales* (1)	*Melaspileaceae* (2)	*Encephalographa*	1
		*Melaspilea*	2
*Filobasidiales* (1)	*Filobasidiaceae* (2)	*Heterocephalacria*	1
		*Zyzygomyces*	1
*Graphidales* (4)	*Diploschistaceae* (2)	*Diploschistes*	7
		*Xalocoa*	1
	*Gomphillaceae* (6)	*Diploschistella*	1
		*Gomphillus*	1
		*Gyalectidium*	1
		*Gyalidea*	1
		*Gyalideopsis*	1
		*Jamesiella*	1
	*Graphidaceae* (2)	*Graphis*	10
		*Phaeographis*	3
	*Thelotremataceae* (1)	*Thelotrema*	3
*Gyalectales* (5)	*Coenogoniaceae* (1)	*Coenogonium*	3
	*Gyalectaceae* (3)	*Gyalecta*	13
		*Neopetractis*	1
		*Ramonia*	2
	*Phlyctidaceae* (1)	*Phlyctis*	2
	*Sagiolechiaceae* (1)	*Sagiolechia*	1
	*Trichotheliaceae* (1)	*Porina*	15
*Helotiales* (2)	*Cordieritidaceae* (4)	*Diplolaeviopsis*	1
		*Llimoniella*	1
		*Rhymbocarpus*	1
		*Unguiculariopsis*	2
	*Pezizellaceae* (1)	*Chalara*	1
*Hypocreales* (4)	*Bionectriaceae* (3)	*Nectriopsis*	2
		*Paranectria*	2
		*Trichonectria*	1
	*Nectriaceae* (2)	*Nectria*	1
		*Xenonectriella*	2
	*Niessliaceae* (1)	*Niesslia*	1
	*Sarocladiaceae* (1)	*Acremonium*	1
*Incertae sedis* (5)	*Harpidiaceae* (1)	*Harpidium*	1
	*Incertae sedis* (14)	*Bachmaniomyces*	1
		*Bachmanniomyces*	1
		*Biatoridium*	1
		*Cyrtidula*	1
		*Endococcus*	1
		*Homostegia*	1
		*Intralichen*	1
		*Lichenodiplis*	1
		*Lichenopuccinia*	1
		*Minutoexcipula*	2
		*Petractis*	1
		*Piccolia*	1
		*Scythioria*	1
		*Wernerella*	1
	*Naetrocymbaceae* (2)	*Leptorhaphis*	2
		*Naetrocymbe*	1
	*Pseudoperisporiaceae* (1)	*Myxophora*	1
	*Strangosporaceae* (1)	*Strangospora*	3
*Lecanorales* (16)	*Catillariaceae* (4)	*Catillaria*	11
		*Placolecis*	1
		*Solenopsora*	7
		*Wadeana*	1
	*Cladoniaceae* (2)	*Cladonia*	78
		*Pycnothelia*	1
	*Haematommataceae* (1)	*Haematomma*	5
		*Catinaria*	1
		*Myochroidea*	2
	*Lecanoraceae* (18)	*Adelolecia*	1
		*Bryonora*	2
		*Carbonea*	4
		*Clauzadeana*	1
		*Glaucomaria*	2
		*Japewia*	1
		*Japewiella*	1
		*Lecanora*	87
		*Lecidella*	14
		*Miriquidica*	7
		*Myriolecis*	8
		*Polyozosia*	1
		*Protoparmeliopsis*	4
		*Pyrrhospora*	2
		*Rhizoplaca*	2
		*Straminella*	1
		*Traponora*	1
		*Tylothallia*	1
	*Megalariaceae* (1)	*Megalaria*	3
	*Parmeliaceae* (39)	*Alectoria*	2
		*Arctoparmelia*	1
		*Brodoa*	2
		*Bryoria*	3
		*Bulbothrix*	1
		*Canoparmelia*	1
		*Cetraria*	8
		*Cetrariella*	1
		*Cetrelia*	2
		*Cornicularia*	1
		*Crespoa*	1
		*Evernia*	2
		*Flavoparmelia*	2
		*Hypogymnia*	4
		*Hypotrachyna*	11
		*Imshaugia*	2
		*Letharia*	1
		*Lethariella*	1
		*Lichen*	5
		*Melanelia*	3
		*Melanelixia*	5
		*Melanohalea*	5
		*Menegazzia*	1
		*Montanelia*	3
		*Nephromopsis*	2
		*Parmelia*	19
		*Parmelina*	4
		*Parmeliopsis*	2
		*Parmotrema*	14
		*Phacopsis*	1
		*Platismatia*	1
		*Protoparmelia*	3
		*Pseudephebe*	1
		*Pseudevernia*	2
		*Punctelia*	7
		*Squamaria*	1
		*Usnea*	36
		*Vulpicida*	1
		*Xanthoparmelia*	16
	*Pilocarpaceae* (6)	*Aquacidia*	2
		*Byssoloma*	4
		*Fellhanera*	2
		*Fellhaneropsis*	1
		*Micarea*	21
		*Schadonia*	1
	*Psilolechiaceae* (1)	*Psilolechia*	1
	*Psoraceae* (5)	*Brianaria*	1
		*Glyphopeltis*	1
		*Protoblastenia*	2
		*Psora*	4
		*Psorula*	1
	*Ramalinaceae* (18)	*Bacidia*	18
		*Bacidina*	11
		*Bellicidia*	1
		*Biatora*	2
		*Bibbya*	1
		*Bilimbia*	3
		*Cliostomum*	3
		*Herteliana*	1
		*Kiliasia*	2
		*Lecania*	27
		*Mycobilimbia*	3
		*Phyllopsora*	1
		*Ramalina*	40
		*Scutula*	2
		*Thalloidima*	10
		*Toninia*	8
		*Toniniopsis*	4
		*Waynea*	5
	*Ramboldiaceae* (1)	*Ramboldia*	4
	*Scoliciosporaceae* (1)	*Scoliciosporum*	5
	*Sphaerophoraceae* (3)	*Bunodophoron*	1
		*Gilbertaria*	1
		*Sphaerophorus*	2
	*Stereocaulaceae* (4)	*Hertelidea*	1
		*Lepraria*	12
		*Squamarina*	7
		*Stereocaulon*	8
	*Tephromelataceae* (3)	*Calvitimela*	1
		*Mycoblastus*	1
		*Tephromela*	3
*Lecideales* (2)	*Lecideaceae* (11)	*Bellemerea*	2
	*Lecideaceae* (11)	*Bryobilimbia*	2
		*Cecidonia*	2
		*Clauzadea*	5
		*Immersaria*	2
		*Lecidea*	44
		*Lecidoma*	1
		*Porpidia*	7
		*Porpidinia*	1
		*Romjularia*	1
		*Stenhammarella*	1
	*Lopadiaceae* (1)	*Lopadium*	1
*Leotiales* (1)	*Mniaeciaceae* (1)	*Epithamnolia*	1
*Leprocaulales* (1)	*Leprocaulaceae* (2)	*Halecania*	2
		*Leprocaulon*	1
*Lichenoconiales* (1)	*Lichenoconiaceae* (1)	*Lichenoconium*	4
*Lichenotheliales* (1)	*Lichenotheliaceae* (2)	*Lichenostigma*	4
		*Lichenothelia*	1
*Lichinales* (3)	*Gloeoheppiaceae* (1)	*Gloeoheppia*	1
	*Lichinaceae* (18)	*Anema*	2
		*Cryptothele*	1
		*Ephebe*	1
		*Heppia*	4
		*Lemmopsis*	1
		*Lempholemma*	3
		*Lichina*	2
		*Lichinella*	3
		*Peccania*	3
		*Phylliscum*	1
		*Porocyphus*	2
		*Psorotichia*	7
		*Pterygiopsis*	1
		*Pyrenocarpon*	1
		*Pyrenopsis*	8
		*Synalissa*	2
		*Thallinocarpon*	1
		*Watsoniomyces*	1
	*Peltulaceae* (1)	*Peltula*	10
*Microthyriales* (1)	*Microthyriaceae* (1)	*Lichenopeltella*	4
*Monoblastiales* (1)	*Monoblastiaceae* (3)	*Acrocordia*	4
		*Anisomeridium*	4
		*Microthelia*	1
*Mycocaliciales* (1)	*Mycocaliciaceae* (4)	*Chaenothecopsis*	2
		*Mycocalicium*	2
		*Phaeocalicium*	2
		*Sphinctrina*	4
*Mycosphaerellales* (3)	*Cystocoleaceae* (1)	*Cystocoleus*	1
		*Sphaerellothecium*	2
		*Stigmidium*	2
	*Teratosphaeriaceae* (1)	*Xanthoriicola*	1
*Mytilinidiales* (1)	*Mytilinidiaceae* (1)	*Taeniolella*	2
*Ostropales* (5)	*Gomphillaceae* (1)	*Corticifraga*	1
	*Graphidaceae* (5)	*Allographa*	3
		*Glyphis*	1
		*Graphina*	1
		*Leucodecton*	1
		*Sanguinotrema*	1
	*Odontotremataceae* (1)	*Skyttea*	2
	*Spirographaceae* (1)	*Spirographa*	1
	*Stictidaceae* (5)	*Absconditella*	1
		*Cryptodiscus*	1
		*Ingvariella*	1
		*Thelopsis*	2
		*Topelia*	1
*Peltigerales* (8)	*Coccocarpiaceae* (2)	*Coccocarpia*	1
		*Spilonema*	2
	*Collemataceae* (11)	*Blennothallia*	1
		*Callome*	1
		*Collema*	9
		*Enchylium*	7
		*Epiphloea*	1
		*Lathagrium*	5
		*Leptogium*	17
		*Paracollema*	1
		*Pseudoleptogium*	1
		*Rostania*	2
		*Scytinium*	20
	*Koerberiaceae* (2)	*Koerberia*	1
		*Tingiopsidium*	1
	*Massalongiaceae* (3)	*Leptochidium*	1
		*Massalongia*	1
		*Polychidium*	1
	*Pannariaceae* (9)	*Erioderma*	1
		*Fuscopannaria*	6
		*Nevesia*	1
		*Pannaria*	4
		*Parmeliella*	3
		*Pectenia*	3
		*Protopannaria*	1
		*Psoroma*	1
		*Staurolemma*	1
	*Peltigeraceae* (7)	*Crocodia*	1
		*Lobaria*	2
		*Lobarina*	1
		*Nephroma*	5
		*Peltigera*	21
		*Ricasolia*	3
		*Sticta*	7
	*Placynthiaceae* (1)	*Placynthium*	7
	*Vahliellaceae* (1)	*Vahliella*	3
*Pertusariales* (6)	*Icmadophilaceae* (3)	*Dibaeis*	1
		*Icmadophila*	1
		*Siphula*	1
	*Megasporaceae* (5)	*Aspicilia*	8
		*Aspiciliella*	2
		*Circinaria*	6
		*Lobothallia*	3
		*Megaspora*	2
	*Ochrolechiaceae* (1)	*Ochrolechia*	14
	*Pertusariaceae* (1)	*Pertusaria*	28
	*Varicellariaceae* (1)	*Varicellaria*	3
	*Variolariaceae* (1)	*Lepra*	15
*Phyllachorales* (1)	*Phyllachoraceae* (1)	*Lichenochora*	1
*Pleosporales* (10)	*Arthopyreniaceae* (2)	*Arthopyrenia*	8
		*Mycomicrothelia*	1
	*Cryptocoryneaceae* (1)	*Cryptocoryneum*	1
	*Dacampiaceae* (2)	*Dacampia*	1
		*Polycoccum*	2
	*Didymosphaeriaceae* (1)	*Didymosphaeria*	1
		*Milospium*	1
		*Refractohilum*	1
		*Vouauxiella*	1
		*Vouauxiomyces*	1
	*Mycoporaceae* (1)	*Mycoporum*	1
		*Naetrocymbe*	1
		*Tomasellia*	1
	*Phaeosphaeriaceae* (1)	*Didymocyrtis*	3
	*Pleomassariaceae* (1)	*Peridiothelia*	2
	*Pyrenidiaceae* (1)	*Pyrenidium*	1
*Pyrenulales* (1)	*Pyrenulaceae* (2)	*Anthracothecium*	1
		*Pyrenula*	10
*Rhizocarpales* (1)	*Rhizocarpaceae* (3)	*Catolechia*	1
		*Epilichen*	1
		*Rhizocarpon*	34
*Sarrameanales* (1)	*Sarrameanaceae* (1)	*Loxospora*	1
*Schaereriales* (1)	*Schaereriaceae* (1)	*Schaereria*	3
*Sclerococcales* (2)	*Dactylosporaceae* (1)	*Sclerococcum*	1
	*Sclerococcaceae* (1)	*Sclerococcum*	4
*Sordariales* (2)	*Helminthosphaeriaceae* (1)	*Endophragmiella*	1
	*Incertae sedis* (3)	*Reconditella*	1
		*Roselliniella*	1
		*Roselliniopsis*	1
*Sporastatiales* (1)	*Sporastatiaceae* (1)	*Sporastatia*	1
*Strigulales* (1)	*Strigulaceae* (3)	*Dichoporis*	3
		*Strigula*	1
		*Swinscowia*	2
*Teloschistales* (2)	*Megalosporaceae* (1)	*Megalospora*	1
	*Teloschistaceae* (33)	*Athallia*	4
		*Blastenia*	9
		*Calogaya*	6
		*Caloplaca*	32
		*Cerothallia*	1
		*Coppinsiella*	1
		*Flavoplaca*	8
		*Gyalolechia*	6
		*Haloplaca*	1
		*Huneckia*	1
		*Ikaeria*	1
		*Kuettlingeria*	7
		*Lendemeriella*	3
		*Leproplaca*	2
		*Marchantiana*	1
		*Olegblumia*	1
		*Opeltia*	1
		*Parvoplaca*	1
		*Pisutiella*	2
		*Polycauliona*	2
		*Pyrenodesmia*	3
		*Rufoplaca*	2
		*Sanguineodiscus*	2
		*Seawardiella*	1
		*Seirophora*	2
		*Solitaria*	1
		*Squamulea*	1
		*Teloschistes*	2
		*Usnochroma*	1
		*Variospora*	5
		*Xanthocarpia*	5
		*Xanthomendoza*	2
		*Xanthoria*	5
*Thelenellales* (1)	*Thelenellaceae* (1)	*Thelenella*	3
*Tremellales* (2)	*Bulleraceae* (1)	*Biatoropsis*	1
	*Tremellaceae* (1)	*Tremella*	5
*Trypetheliales* (1)	*Trypetheliaceae* (1)	*Julella*	3
*Umbilicariales* (4)	*Fuscideaceae* (3)	*Fuscidea*	6
		*Maronea*	1
		*Orphniospora*	1
	*Ophioparmaceae* (2)	*Hypocenomyce*	1
		*Ophioparma*	1
	*Ropalosporaceae* (1)	*Ropalospora*	1
	*Umbilicariaceae* (2)	*Umbilicaria*	19
		*Xylopsora*	1
*Verrucariales* (2)	*Incertae sedis* (1)	*Botryolepraria*	1
	*Verrucariaceae* (29)	*Agonimia*	5
		*Anthracocarpon*	1
		*Bagliettoa*	8
		*Catapyrenium*	3
		*Clavascidium*	2
		*Dermatocarpon*	4
		*Endocarpon*	4
		*Heteroplacidium*	2
		*Hydropunctaria*	1
		*Involucropyrenium*	1
		*Merismatium*	1
		*Muellerella*	4
		*Nesothele*	2
		*Normandina*	2
		*Parabagliettoa*	2
		*Phaeospora*	1
		*Phylloblastia*	1
		*Placidiopsis*	2
		*Placidium*	8
		*Placocarpus*	1
		*Placopyrenium*	2
		*Polyblastia*	3
		*Psoroglaena*	1
		*Staurothele*	2
		*Telogalla*	1
		*Thelidium*	2
		*Verrucaria*	37
		*Verruculopsis*	2
		*Wahlenbergiella*	2
*Vezdaeales* (1)	*Vezdaeaceae* (1)	*Vezdaea*	2
*Xylariales* (1)	*Leptosilliaceae* (1)	*Leptosillia*	1

**Table 2:** Overview of the distribution of taxa and occurrences per province. The table includes the number of occurrences (Occurrences), taxonomic richness (Taxa), rate of taxa in the province in relation to the total in the country (% Taxa), number of taxa with only on occurrence in the province (Singletons), number of references contributing with occurrences for the province (Number of References), the highest number of taxa referred to in a single reference (Maximum Taxa in a Single Reference) and the Simpson index for the number of taxa in references (Simpson_D). In the counts of taxa, only valid names (accepted names and synonyms) are considered.

Region	Occurrences	Taxa	% Taxa	Singletons	Number of References	Maximum Taxa in a Single Reference	Simpson_D
Portugal	43,718	2012	100	388	381	826	0.020
Province							
Estremadura	7849	1071	53	303	201	472	0.043
Minho	6158	689	34	158	146	351	0.052
Algarve	5460	872	43	214	155	691	0.087
Beira Alta	5169	627	31	139	134	313	0.057
Baixo Alentejo	4174	395	20	126	100	155	0.067
Alto Alentejo	3676	435	22	108	87	244	0.096
Trás-os-Montes e Alto Douro	3660	792	39	252	106	418	0.078
Beira Litoral	2695	596	30	217	122	233	0.052
Douro Litoral	2358	451	22	115	97	219	0.086
Ribatejo	894	187	9	64	48	99	0.129
Beira Baixa	612	228	11	91	36	125	0.143

## Data Availability

The full checklist dataset is published and openly accessible through GBIF at https://doi.org/10.15468/chense [35]. In addition, further data treatments are included in the article/Supplementary Materials. Code created during this study is available at https://github.com/rpfigueira/lichen-checklist-pt (accessed on 16 June 2026).

## Supplementary Materials

The following supporting information can be downloaded at: https://www.mdpi.com/article/10.3390/jof12080549/s1, Table S1: Bibliographic references used as sources for lichen occurrences in Mainland Portugal; Table S2: Tables and terms included in the tables of the Darwin Core standardised dataset; Table S3: Bibliographic references used as taxonomic sources for lichen names of occurrences in Mainland Portugal; Table S4: Taxa excluded from Mainland Portugal after critical assessments of occurrences; Table S5: Taxa recorded only once in Mainland Portugal, representing the single Continental European occurrences.
