# Supplementary material for: Checklist of Lichens and Lichenicolous Fungi from Mainland Portugal
Source: J Fungi (Basel). 2026 Jul 23;12(8):549. doi: 10.3390/jof12080549 (PMC13514346; doi:10.3390/jof12080549)
Supplement: Supplementary file 1 [file jof-12-00549-s001.zip › Table_S5.pdf]

Table S5: Taxa recorded only once in Mainland Portugal, representing the single Continental European occurrences.

Family	Genus	Taxon	Accepted name
Caliciaceae	*Sculptolumina*	*Sculptolumina japonica* (Tuck.) Marbach	
Chrysotrichaceae	*Chrysothrix*	*Chrysothrix xanthina* (Vain.) Kalb	
Gomphillaceae	*Gyalectidium*	*Gyalectidium minus* Sérus.	
Graphidaceae	*Graphis*	*Graphis cincta* (Pers.) Aptroot	
Graphidaceae	*Graphis*	*Graphis crebra* Vain.	
Graphidaceae	*Graphis*	*Graphis handelii* Zahlbr.	
Graphidaceae	*Graphis*	*Graphis leptospora* Vain.	*Allographa leptospora* (Vain.) Lücking & Kalb
Graphidaceae	*Graphis*	*Graphis lineola* Ach.	
Graphidaceae	*Graphis*	*Graphis plumierae* Vain.	
Graphidaceae	*Graphis*	*Graphis verminosa* Müll. Arg.	*Allographa verminosa* (Müll. Arg.) Lücking & Kalb
Lecanoraceae	*Lecanora*	*Lecanora sabinae* Hern.-Padr. & Vänskä	
Microthyriaceae	*Lichenopeltella*	*Lichenopeltella heppiae* van den Boom	
Microthyriaceae	*Lichenopeltella*	*Lichenopeltella physciae* Diederich	
Parmeliaceae	*Canoparmelia*	*Canoparmelia aptata* (Kremp.) Elix & Hale	
Parmeliaceae	*Parmotrema*	*Parmotrema grayanum* (Hue) Hale	
Peltulaceae	*Peltula*	*Peltula farinosa* Büdel	
Pezizellaceae	*Chalara*	*Chalara ramalinae* Etayo	
Physciaceae	*Physcia*	*Physcia undulata* Moberg	
Pilocarpaceae	*Fellhanera*	*Fellhanera seroexspectata* Sérus.	
Ramalinaceae	*Bacidina*	*Bacidina brittoniana* (Riddle) LaGreca & S. Ekman	
Ramalinaceae	*Lecania*	*Lecania juniperi* van den Boom	
Ramalinaceae	*Ramalina*	*Ramalina huei* Harm.	
Ramalinaceae	*Waynea*	*Waynea algarvensis* van den Boom	
Sagiolechiaceae	*Sagiolechia*	*Sagiolechia atlantica* Henssen	
Teloschistaceae	*Caloplaca*	*Caloplaca squamulosa* (Wedd.) B. de Lesd.	
Teloschistaceae	*Caloplaca*	*Caloplaca stanfordensis* H. Magn.	
Teloschistaceae	*Ikaeria*	*Ikaeria serusiauxii* Sipman	
Thelotremataceae	*Thelotrema*	*Thelotrema laurisilvae* Lücking & Breuss	
Trapeliaceae	*Trapeliopsis*	*Trapeliopsis gymnidiata* Aptroot & Schumm	
