# Supplementary material for: Checklist of Lichens and Lichenicolous Fungi from Mainland Portugal
Source: J Fungi (Basel). 2026 Jul 23;12(8):549. doi: 10.3390/jof12080549 (PMC13514346; doi:10.3390/jof12080549)
Supplement: Supplementary file 1 [file jof-12-00549-s001.zip › Table_S2.pdf]

Table S2: Tables and terms included in the tables of the Darwin Core standardised dataset.

DwC table	DwC Term	Original column	Notes
Taxon (core)	taxonID		Global unique identifier generated in OpenRefine
Taxon (core)	accept-edNameUsageID		Global unique identifier generated in OpenRefine
Taxon (core)	scientificName	species_normalised	
Taxon (core)	acceptedNameUsage	species_current_name	
Taxon (core)	nameAccordingTo		
Taxon (core)	kingdom	kingdom	
Taxon (core)	phylum	phylum	
Taxon (core)	class	class	
Taxon (core)	order	order	
Taxon (core)	family	family	
Taxon (core)	genus	species_normalised	original value parsed to ex-tract genus
Taxon (core)	specificEpithet	species_normalised	original value parsed to ex-tract specific epithet
Taxon (core)	infraspecificEpithet	species_normalised	original value parsed to ex-tract infraspecific epithet
Taxon (core)	taxonRank		
Taxon (core)	scientificNameAuthor-ship	species_normalised	original value parsed to ex-tract authorship
Taxon (core)	taxonomicStatus	taxonomicStatus	
Taxon (core)	nomenclaturalStatus	nomenclaturalStatus	
Taxon (core)	taxonRemarks	Remarks	
Occurrence	basisOfRecord		
Occurrence	occurrenceID		Global unique identifier generated in OpenRefine
Occurrence	recordedBy	recordedBy / identi-fiedBy (Leg/det)	original value parsed to ex-tract collector
Occurrence	occurrenceStatus		
Occurrence	associatedReferences	publication	
Occurrence	occurrenceRemarks	remarks	original value processed
Occurrence	eventDate	sampling_date	
Occurrence	habitat	ecology	
Occurrence	country		
Occurrence	countryCode		
Occurrence	stateProvince		
Occurrence	municipality		
Occurrence	locality	local	
Occurrence	minimumElevation-InMeters	altitude	
Occurrence	maximumElevation-InMeters	altitude	
Occurrence	verbatimElevation	altitude	
Occurrence	decimalLatitude	Latitude	
Occurrence	decimalLongitude	Longitude	

Occurrence	coordinateUncertaintyInMeters		
Occurrence	coordinatePrecision		
Occurrence	footprintWKT		
Occurrence	georeferenceSources		
Occurrence	verbatimIdentification	species_source	
Occurrence	identificationQualifier	identificationQualifier	
Occurrence	typeStatus		
Occurrence	identifiedBy	recordedBy / identifiedBy (Leg/det)	original value parsed to extract determiner
Occurrence	identificationRemarks	comments_notes_identification	
Occurrence	taxonID		
Occurrence	acceptedNameUsageID		
Occurrence	scientificName		
Occurrence	acceptedNameUsage		
Occurrence	kingdom	kingdom	
Occurrence	phylum	phylum	
Occurrence	class	class	
Occurrence	order	order	
Occurrence	family	family	
Occurrence	genus	species_normalised	original value parsed to extract genus
Occurrence	specificEpithet	species_normalised	original value parsed to extract specific epithet
Occurrence	infraspecificEpithet	species_normalised	original value parsed to extract infraspecific epithet
Occurrence	taxonRank		
Occurrence	scientificNameAuthorship	species_normalised	original value parsed to extract authorship
Occurrence	taxonomicStatus		
Occurrence	nomenclaturalStatus		
Occurrence	taxonRemarks		
Distribution	locationID		Wikidata ID added, obtained by reconcile to Wikidata type “former Portuguese province” (Q2363637), using OpenRefine.
Distribution	locality	province	Provinces from administrative regions of 1936, not currently in use
Distribution	countryCode		
Distribution	occurrenceStatus		
Distribution	eventDate		
Distribution	source		
ExtendedMeasurementOrFact	measurementID		
ExtendedMeasurementOrFact	occurrenceID		
ExtendedMeasurementOrFact	measurementType		added substrate as a property

ExtendedMeasurementOrFact	measurementValue		added the substrat value, using the following vocabulary: artificial, corticolous, foliicolous, lichenicolous, lignicolous, muscicolous, saxicolous, terricolous
ExtendedMeasurementOrFact	measurementDeterminedDate	sampling_date	
ExtendedMeasurementOrFact	measurementDeterminedBy	recordedBy / identifiedBy (Leg/det)	original value parsed to extract collector
ResourceRelationship	resourceRelationshipID		
ResourceRelationship	resourceID		
ResourceRelationship	relatedResourceID		
ResourceRelationship	relationshipOfResource		added value “duplicate of” to identify two citation occurrences that correspond to the same specimen collected in the field
