# Supplementary material for: Checklist of Lichens and Lichenicolous Fungi from Mainland Portugal
Source: J Fungi (Basel). 2026 Jul 23;12(8):549. doi: 10.3390/jof12080549 (PMC13514346; doi:10.3390/jof12080549)
Supplement: Supplementary file 1 [file jof-12-00549-s001.zip › Table_S3.pdf]

Table S3: Bibliographic references used as taxonomic sources for lichen names of occurrences in Mainland Portugal.

Amrani, S., Seaward, M. R. D., Sipman, H. J. M. & Feuerer, T. (2018). Lichenological exploration of Algeria II: checklist of lichenized, lichenicolous and allied fungi. Herzogia, 31, 817-892. https://doi.org/10.13158/heia.31.2.2018.817

Aptroot, A. (2006). Three new species of *Lithothelium* (*Pyrenulaceae*) from China and Thailand, with a revised world key and annotated list of species. Lichenologist, 38(6), 541-548.

Aptroot, A. (2011). A world key to the species of *Anthracothecium* and *Pyrenula*. The Lichenologist, 44 (1), 5–53.

Araujo Caviró, E. (2016). Sistemática integrada del género "*Usnea*" Dill. "Ex" Adans: ("*Parmeliaceae*) en la Península Ibérica. [Thesis]

Arcadia, L. (2024). List of lichen names. Version (05-Nov-2024) from: www.lichensofgreece.com

Arup, U., Bertrand, M., Navarro-Rosinés, P., Nimis, P. L., Roux, C. & Søchting, U. (2023). Taxonomy and nomenclature of a much-misunderstood Mediterranean lichen, *Caloplaca subochracea* auct. (Lichenized Ascomycota, *Teloschistaceae*). Borziana, 4, 33-49.

Burgaz, A. R., Ahti, T. & Pino-Bodas, R. (2020). Mediterranean *Cladoniaceae*. Spanish Lichen Society, 122 pp.

Carballal, R., López de Silanes, M. E., Paz-Bermúdez, G. & Álvarez, J. (2004) *Ostropales*: *Graphidaceae*, *Solorinellaceae*; *Gyalectales*: *Gyalectaceae*. Flora Liquenológica Ibérica, 2, 1–48.

Carballal, R., Paz-Bermúdez, G., López de Silanes, M. E. & Pérez Valcárcel, C. (2010). *Pannariaceae*. Flora Liquenológica Ibérica, 6. Sociedad Española de Liquenología, Pontevedra.44 pp.

Ceynowa-Giełdon, M. & Adamska, E. (2014). Notes on the genus *Thelidium* (*Verrucariaceae*, lichenized Ascomycota) in the Kujawy region (north-central Poland). Ecological Questions. Online. 20 August 2014. Vol. 19, pp. 25-33. [Accessed 2024]

Clerc, P. (2011). Notes on the genus *Usnea* Adanson lichenized Ascomycota. III. Bibliotheca Lichenologica, 106, 41-51.

Consortium of Lichen Herbaria (2025) http//lichenportal.org/portal/index.php. Accessed on 2025-01

Diederich, P., Lawrey, J. D. & Ertz, D. (2018). The 2018 classification and checklist of lichenicolous fungi, with 2000 non-lichenized, obligately lichenicolous taxa. Bryologist, 121(3), 340-425.

Ekman, S., Tønsberg, T. & Jørgensen, P. M. (2019). The *Sticta fuliginosa* group in Norway and Sweden. Graphis Scripta, 31(4), 23–33.

Ertz, D. & Tehler, A. (2011) The phylogeny of *Arthoniales* (Pezizomycotina) inferred from nucLSU and RPB2 sequences. Fungal Diversity, 49, 47–71.

Frisch, A., Thor, G., Ertz, D. & Grube, M. (2014) The Arthonialean challenge: Restructuring *Arthoniaceae*. Taxon, 63(4), 727-744.

GBIF.org (2024). GBIF Home Page. Available from: https://www.gbif.org

Giralt, M. (2010). *Physciaceae* I. *Endohyalina*, *Rinodina* y *Rinodinella*. Flora Liquenológica Ibérica, 5. Barcelona, 105 pp.

Gray, S. F. (1821). A Natural Arrangement of British Plants, 1. 824 pp.

Hale, M. E. (1990). A synopsis of the lichen genus *Xanthoparmelia* (Vainio) Hale (Ascomycotina, *Parmeliaceae*). Smithsonian Contributions to Botany, 74, 1–250.

Hale, M. E. (1987). A monograph of the lichen genus *Parmelia* Acharius sensu stricto (Ascomycotina: *Parmeliaceae*). Smithsonian Contributions to Botany, 66, 1–54.

Hawksworth, D. L., Blanco, O., Divakar, P. K., Ahti, T. & Crespo, A (2008). A first checklist of parmelioid and similar lichens in Europe and some adjacent territories, adopting revised generic circumscriptions and with indications of species distributions. The Lichenologist, 40(1), 1-21.

Index Fungorum (2024). Index Fungorum Page. Available from: www.indexfungorum.org

Jørgensen, P. M. (2003). Conspectus familiae *Pannariaceae* (Ascomycetes lichenosae). Universitetet i Bergen, Botanisk Institutt, 79 pp.

Jørgensen, P. M. (1994). Further notes on European taxa of the lichen genus Leptogium, with emphasis on the small species. The Lichenologist, 26: 1–29.

Jørgensen, P. M. (2007). *Collemataceas*. In: Nordic Lichen Flora. Volume 3. Cyanolichens. Ahti, T., Jørgensen, P.M., Kristinsson, H., Moberg, R., Søchting, U., Thor, G., Eds.; Nordic Lichen Society: Uddevalla, Sweden, pp. 14–42.

Kirk, P. M. (2025). Species Fungorum Plus (version Apr 2024). In: O. Bánki, Y. Roskov, M. Döring, et al., Catalogue of Life (2025-10-10 XR). Catalogue of Life Foundation, Amsterdam, Netherlands. https://doi.org/10.15468/ts7wsb

Knezevic, K. & Mayrhofer, H. (2009). Catalogue of the lichenized and lichenicolous fungi of Montenegro. Phyton (Horn, Austria), 48(2), 283-328.

Kurokawa, S. (1962). A monograph of the genus *Anaptychia*. Beihefte zur Nova Hedwigia, 6, 1–115.

Lawrey, J. D. & P. Diederich (2018). Lichenicolous fungi – worldwide checklist, including isolated cultures and sequences available URL: http://www.lichenicolous.net

Llimona, X. & Hladun, N. L. (2001). Checklist of the lichens and lichenicolous fungi of the Iberian Peninsula and Balearic Islands. Bocconea, 14, 1-581.

Mycobank (2023-2024). Available from: https://www.mycobank.org/page/Name%20details%20page/field/Mycobank%20%23/403171

Nimis, P. L. (1993). The Lichens of Italy. Museo Regionale di Scienze Naturali, Torino. 897 pp.

Nimis, P. L. (2024). ITALIC - The Information System on Italian Lichens. Version 8.0. University of Trieste, Dept. of Biology, (https://dryades.units.it/italic), accessed on 2004

Nordin, A., Savić, S. & Tibell, L. (2010). Phylogeny and taxonomy of *Aspicilia* and *Megasporaceae*. Mycologia, 102(6), 1339–1349.

Nylander, N. (1888). Lichens du nord du Portugal. Boletim da Sociedade Broteriana, 6, 198-.249.

Paz-Bermúdez , G. & López de Silanes , M. E. (2007). The identity of two lichen species described from Portugal by J. Harmand. The Bryologist, 110(1), 119-122.

Paz-Bermúdez, G. (2003). The identity of some lichens described from Portugal by G. Sampaio and W. Nylander. Nova Hedwigia, 77(1-2), 115-123.

Paz-Bermúdez, G., Aguiar-Branco, H. & Folhadela, E. (2002). Typification of names of lichen taxa described by G. Sampaio and some others, deposited in Porto Herbarium (PO). Taxon, 51, 771-785.

Paz-Bermúdez, G., Carballal, R. & Valcárcel, C. P. (2006). *Caloplaca peregrina* Samp. (*Teloschistaceae*), a synonym of *C. pellodella* (Nyl.) Hasse. Bryologist, 109(2), 185-187.

Pino-Bodas, R., Ahti, T., Stenroos, S., Martín, M. & Burgaz, A. R. (2013). Multilocus approach to species recognition in the *Cladonia humilis* complex (*Cladoniaceae*, Ascomycota). American Journal of Botany, 100(4), 664-78.

Roca Valiente, B., Hawksworth, D., Pérez-Ortega, S., Sancho, L. & Crespo, A (2016). Type studies in the *Rhizocarpon geographicum* group (*Rhizocarpaceae*, lichenized Ascomycota). Lichenologist, 48, 97–110.

Roux, C., et at. (2017). Catalogue des Lichens et Champignons Lichénicoles de France Métropolitaine, 2nd ed.; Association Française de Lichénologie: Fontainebleau, France, 1581pp

Schultz, M., Wedin, M., Diel, H. & Prieto, M. (2015). *Epiphloea* belongs to *Collemataceae* (Lecanoromycetes, lichenized Ascomycota). Lichenologist, 47(6), 369–378.

Suija, A., Hawksworth, D. L. & Pérez-Ortega, S. (2018). The generic name *Abrothallus* (*Abrothallales*, Dothideomycetes), and names proposed in the genus by Giuseppe De Notaris, Søren Christian Sommerfelt, and Ignaz Kotte. Taxon, 67(6), 1169-1179.

Tehler, A., Dahlkild, Å., Eldenäs, P. & Feige, G. B. (2004). The phylogeny and taxonomy of Macaronesian, European and Mediterranean *Roccella* (*Roccellaceae*, *Arthoniales*). Acta Univ. Ups. Symb. Bot. Ups, 34(1), 405–428. Uppsala. ISBN 91-554-6025-9.

Tehler, A., Ertz, D. & Irestedt, M. (2013) The genus *Dirina* (*Roccellaceae*, *Arthoniales*) revisited. The Lichenologist, 45(4), 427-476.

Torrente P. & Egea J. M. (1991). *Llimonaea*, a new genus of lichenized fungi in the order *Opegraphales* (Ascomycotina). Nova Hedwigia, 52, 239-245.

Turney, S. (2014). Catalog of lichens, lichenicolous and allied fungi in California (second revision) https://ucjeps.berkeley.edu/constancea/85/tucker.html
