# Supplementary material for: Checklist of Lichens and Lichenicolous Fungi from Mainland Portugal
Source: J Fungi (Basel). 2026 Jul 23;12(8):549. doi: 10.3390/jof12080549 (PMC13514346; doi:10.3390/jof12080549)
Supplement: Supplementary file 1 [file jof-12-00549-s001.zip › Table_S1.pdf]

Table S1: Bibliographic references used as sources for lichen occurrences in Mainland Portugal.

Abbayes, H. des (1959). L'aire geographique de *Cladonia mediterranea* Duv. et des Abb. (lichens) et son ecologie d'apres de nouvelles observations. Revue Bryologique et Lichénologique, 28(3–4), 355–358.

Ahti, T. (1961). Taxonomic studies on reindeer lichens (*Cladonia*, subgenus *Cladina*). Annales Botanici Societatis Zoologicae Botanicae Fennicae "Vanamo," 32(1), 1–160. https://books.google.pt/books?id=rBHIzwEACAAJ

Aires-Barros, L. (1991). New data on saxicolous lichenic vegetation in monuments as bioindicator of pollution. La conservation des monuments dans le bassin méditerranéen, 115–120. SYRACUSE. https://bibliotheques.inp.fr/Default/doc/SYRACUSE/404285/new-data-on-saxicolous-lichenic-vegetation-in-monuments-as-bioindicator-of-pollution

Aires-Barros, L., Basto, M. J., Graça, R. C., Alves, L. M., Esteves, L., Carmo, A. & Ribeiro, I. (1990). The decay of limestone caused by saxicolous lichens: the case of the Monastery of Jerónimos, Lisbon, Portugal. In: The conservation of monuments in the Mediterranean Basin: the influence of coastal environment and salt spray on limestone and marble. Proceedings of the 1st International Symposium, Bari, 7-10 June 1989 (pp. 225–229). Grafo.

Almborn, O. (1942). Lichenological notes. Botaniska Notiser, 2, 387-403.

Alonso, F. L. & Egea, J. M. (1995). Líquenes calcícolas y terrícolas de algunas localidades costeras de Portugal. Nova Acta Científica Compostelana (Bioloxía), 5, 39–48.

Alonso, F. & Egea, J. (1994). Algunos líquenes interesantes de áreas costeras del sur de la Península Ibérica y Marruecos. Cryptogamie, Bryologie-Lichénologie, 15(3), 225–238.

Alonso, F. L. & Egea, J. M. (1994). Sobre las comunidades de liquenes calcicolas de zonas costeras del Sur de la Peninsula Iberica y Marruecos. Studia Geobotanica, 14, 3–25. http://hdl.handle.net/10077/15232

Amo de Paz, G., Lumbsch, H. T., Cubas, P., Elix, J. A. & Crespo, A. (2010). The genus *Karoowia* (*Parmeliaceae*, Ascomycota) includes unrelated clades nested within *Xanthoparmelia*. Australian Systematic Botany, 23(3), 173–184. https://doi.org/10.1071/SB09055

Amo y Mora, M. (1870). Flora cryptogámica de la Península Ibérica, que contiene la descripcion de las plantas acotyledóneas que crecen en España y Portugal, distribuidas segun el método de familias. Imprenta de D. Indalecio Ventura.

Andrés, J. A. (2003). The genus *Pachyphiale* (*Gyalectales*, Ascomycotina) in the Iberian Peninsula. Nova Hedwigia, 77(1–2), 139–146. https://doi.org/10.1127/0029-5035/2003/0077-0139

Andrés, J. A. & Caraballal Durán, R. (2001). The genus *Dimerella* (*Gyalectales*, Ascomycotina) in Peninsular Spain and Portugal. Nova Hedwigia, 73(3–4), 409–418. https://doi.org/10.1127/nova.hedwigia/73/2001/409

Andrés, J. A. & López de Silanes, M. (2002). The genus *Gyalecta* (*Gyalectales*, Ascomycotina) in the Iberian Peninsula. Nova Hedwigia, 74(1–2), 257–273.

Aptroot, A. & van den Boom, P. P. G. (1995). *Strigula lateralis* spec. nov. with notes on the genus *Julella* (Ascomycetes). Mycotaxon, 56, 1–8.

Aptroot, A., Knaap, W. O. van der & Jansen, J. (1992). Twelve new lichens for Portugal collected from the Serra da Estrela. Cryptogamie, Bryol. Lich, 13(1), 71–73.

Aragón, G., Martínez, I. & Otálora, M. (2004). New data on the distribution of *Leptogium azureum* (Swartz) Mont. The Lichenologist, 36(5), 345–347.

Aragón, G., Otálora, M. A. G. & Martínez, I. (2005). New data on the genus *Leptogium* (lichenized ascomycetes) in the Iberian Peninsula. Nova Hedwigia, 80(1–2), 199–226.

Argüello, A., del Prado, R., Cubas, P. & Crespo, A. (2007). *Parmelina quercina* (*Parmeliaceae*, *Lecanorales*) includes four phylogenetically supported morphospecies. Biological Journal of the Linnean Society, 91(3), 455–467. https://doi.org/10.1111/j.1095-8312.2007.00810.x

Arnold, F. (1868). Lichenes Lusitaniae. Flora (Regensburg), 16, 241–256.

Arroyo Cabeza, R. (1991). El género *Ramalina* Ach. en la Península Ibérica: química, quimiotaxonomía, morfología, anatomía y distribución [Universidad Complutense de Madrid]. https://produccioncientifica.ucm.es/documentos/5d1ffb2d2999521e412dde8d

Arroyo Cabeza, R., Seriñá Ramírez, E. & Araujo, E. (2011). *Ramalina carminae* (Ascomycota: *Ramalinaceae*), a new species from Europe. Botanica Complutensis, 35, 5–14. https://doi.org/10.5209/REV_BOCM.2011.V35.1

Arvidsson, L. (1982). A monograph of the lichen genus *Coccocarpia*. Opera Botanica, 67, 1–96.

Augusto, S., Máguas, C., Matos, J., Pereira, M. J. & Branquinho, C. (2010). Lichens as an integrating tool for monitoring PAH atmospheric deposition: A comparison with soil, air and pine needles. Environmental Pollution, 158(2), 483–489. https://doi.org/10.1016/j.envpol.2009.08.016

Azuaga García, T. & Gómez Bolea, A. (2001). Presència de *Letharia vulpina* (L.) Hue a les Valls d'Andorra. Hàbitats, 2, 30–33.

Baptista, M. S., Teresa Vasconcelos, M., Paulo Cabral, J., Carmo Freitas, M. & Pacheco, A. M. G. (2006). The ability of biological and organic synthetic materials to accumulate atmospheric particulates containing copper, lead, nickel and strontium. Journal of Environmental Monitoring, 8(1), 147–152. https://doi.org/10.1039/B509177M

Barrasa, J. M. & Rico, V. J. (2001). Lichenized species of *Omphalina* (*Tricholomataceae*) in the Iberian Peninsula. The Lichenologist, 33(5), 371–386. https://doi.org/10.1006/lich.2001.0336

Barros, G. J. (1942). Contribuição para o estudo dos líquenes e dos briófitos dos troncos das oliveiras. Agronomia Lusitana, 4(4), 293–303.

Bento-Pereira, F. (1981). Fungos liquenizados e liquenícolas de Portugal. Portugaliae Acta Biologica, 13, 126–139.

Blanco, O., Crespo, A., Divakar, P. K., Elix, J. A. & Lumbsch, H. T. (2005). Molecular phylogeny of parmotremoid lichens (Ascomycota, *Parmeliaceae*). Mycologia, 97(1), 150–159. https://doi.org/10.1080/15572536.2006.11832848

Blom, H. H. & Lindblom, L. (2010). *Degelia cyanoloma* (Schaer.) H. H. Blom & L. Lindblom comb. et stat. nov., a distinct species from western Europe. The Lichenologist, 42(1), 23–27. https://doi.org/10.1017/S002428290999020X

Boqueras, M. & Llimona, X. (2003). The genus *Pertusaria* (lichenised Ascomycotina) on the Iberian Peninsula and Balearic Islands. I. Subgenus *Pertusaria*. Mycotaxon, 88, 471–492.

Boqueras, M., Barbero, M. & Llimona, X. (1999). El género *Ochrolechia* A. Massal. (*Pertusariaceae*, líquenes) en España y Portugal. Cryptogamie, Mycologie, 20, 303–328.

Bouly de Lesdain, M. (1921). Notes lichénologiques XVIII. Bulletin de La Société Botanique de France, 68(2), 203–207.

Branquinho, C., Brown, D. H. & Catarino, F. (1997). The cellular location of Cu in lichens and its effects on membrane integrity and chlorophyll fluorescence. Environmental and Experimental Botany, 38(2), 165–179. https://doi.org/10.1016/S0098-8472(97)00015-4

Branquinho, C., Brown, D. H., Máguas, C. & Catarino, F. (1997). Lead (Pb) uptake and its effects on membrane integrity and chlorophyll fluorescence in different lichen species. Environmental and Experimental Botany, 37(2–3), 95–105. https://doi.org/10.1016/S0098-8472(96)01038-6

Branquinho, C., Catarino, F., Brown, D. H., Pereira, M. J. & Soares, A. (1999). Improving the use of lichens as biomonitors of atmospheric metal pollution. Science of The Total Environment, 232(1), 67–77. https://doi.org/10.1016/S0048-9697(99)00111-4

Branquinho, C., Figueira, R. & Catarino, F. (1994). Líquenes como biomonitores de metais pesados associados a tráfego rodoviário. Actas da 4ª Conferência Nacional Sobre a Qualidade do Ambiente, 3, 175–181.

Breuss, O. (1990). Die Flechtengattung *Catapyrenium* (*Verrucariaceae*) in Europa. Stapfia, 23.

Breuss, O. (1998). Drei neue holz- und borkenbewohnende *Verrucaria*-Arten mit einem Schlüssel der bisher bekannten Taxa. Linzer Biologische Beiträge, 30(2), 831–836. http://www.landesmuseum.at/pdf_frei_remote/LBB_0030_2_0831-0836.pdf

Brotero, F. A. (1804). Flora lusitanica, seu, plantarum, quae in Lusitania vel sponte crescunt, vel frequentius coluntur, ex florum praesertim sexubus systematice distributarum, synopsis, 2. Ex Typographia Regia. https://www.biodiversitylibrary.org/bibliography/45110

Burgaz, A. R. (2009). El género *Cladonia* en la Península Ibérica. Supergrupo *Cocciferae*. Botanica Complutensis, 33, 9–28.

Burgaz, A. R. (2010). Nuevos datos sobre el género *Placynthium* en la Península Ibérica. Botanica Complutensis, 34, 5–19. https://revistas.ucm.es/index.php/BOCM/article/view/BOCM1010110005A

Burgaz, A. R. (2011). *Peltigerales*: *Massalongiaceae* y *Placynthiaceae*. Flora Liquenológica Ibérica, 8. Sociedad Española de Liquenología.

Burgaz, A. R. & Ahti, T. (2009). *Cladoniaceae.* Flora Liquenológica Ibérica, 4. Sociedad Española de Liquenología.

Burgaz, A. R. & Martínez, I. (1995). Aportaciones a la corología del género *Peltigera* Willd. en la Península Ibérica. I. Grupo de *Peltigera canina*. Botanica Complutensis, 20, 19–33. https://revistas.ucm.es/index.php/BOCM/article/view/BOCM9595110019A

Burgaz, A. R. & Martínez, I. (1999). La familia *Lobariaceae* en la Península Ibérica. Botanica Complutensis, 23, 59–90.

Burgaz, A. R. & Martínez, I. (1999). The genus *Nephroma* Ach. in the Iberian Peninsula. Cryptogamie, Mycologie, 20(3), 225–235.

Burgaz, A. R. & Martínez, I. (2001). The genera *Koerberia*, *Leptochidium*, *Massalongia* and *Polychidium* (*Peltigerales sensu lato*) in the Iberian Peninsula. Nova Hedwigia, 73(3–4), 381–392. https://doi.org/10.1127/nova.hedwigia/73/2001/381

Burgaz, A. R. & Martínez, I. (2003). *Peltigerales*: *Lobariaceae*, *Nephromataceae*, *Peltigeraceae*. Flora Liquenológica Ibérica, 1. Sociedad Española de Liquenología.

Burgaz, A. R., Ahti, T. & Carvalho, P. (1999). Contribution to the study of *Cladoniaceae* in Portugal. Portugaliae Acta Biologica, Série B, Sistemática, Ecologia, Biogeografia e Paleontologia, 18, 121–168.

Burgaz, A. R., Rodrígues de Lope, I. & Martínez, I. (2002). The *Placynthium* nigrum group in the Iberian Peninsula. Cryptogamie, Mycologie, 23(4), 367–375.

Cabral, J. P. (2002). Differential sensitivity of four *Lobaria* lichens to copper in vitro. Environmental Toxicology and Chemistry, 21(11), 2468–2476. https://doi.org/10.1002/etc.5620211128

Calatayud, V. & Rico, V. (1999). Chemotypes of *Dimelaena oreina* (Ascomycotina, *Physciaceae*) in the Iberian Peninsula. The Bryologist, 102(1), 39–44.

Calatayud, V., Atienza, V. & Barreno, E. (1995). Lichenicolous fungi from the Iberian Peninsula and the Canary Islands I. Mycotaxon, 55, 363–382.

Canha, N., Freitas, M. C. & Pacheco, A. M. G. (2013). Response of air-pollution biomonitors under three different meteorological conditions. Journal of Radioanalytical and Nuclear Chemistry, 295(1), 489–496. https://doi.org/10.1007/s10967-012-1918-4

Canha, N., Freitas, M. do C. & Almeida, S. M. (2019). Contribution of short irradiation instrumental neutron activation analysis to assess air pollution at indoor and outdoor en-

vironments using transplanted lichens. Journal of Radioanalytical and Nuclear Chemistry, 320(1), 129–137. https://doi.org/10.1007/s10967-019-06461-5

Carballal, R., López de Silanes, M. E., Paz-Bermúdez, G. & Álvarez, J. (2004). *Ostropales*: *Graphidaceae*, *Solorinellaceae*; *Gyalectales*: *Gyalectaceae*. Flora Liquenológica Ibérica, 2. Sociedad Española de Liquenología.

Carballal, R. & López de Silanes, M. E. (2006). Los géneros *Moelleropsis*, *Protopannaria* y *Psoroma* en la Península Ibérica. Cryptogamie, Mycologie, 27(1), 69–77.

Carballal, R. & Paz-Bermúdez, G. (2007). The genera *Coccocarpia* (*Coccocarpiaceae*, Ascomycota), Degelia and *Erioderma* (*Pannariaceae*, Ascomycota), in the Iberian Peninsula. Nova Hedwigia, 85(1–2), 51–62.

Carballal, R., Paz-Bermúdez, G., López de Silanes, M. E. & Pérez Valcárcel, C. (2010). *Pannariaceae*. Flora Liquenológica Ibérica, 6. Sociedad Española de Liquenología.

Carvalho, P. (1997). Flora liquénica do Parque Natural da Serra de S. Mamede. Portugaliae Acta Biologica, Sér. B, Sist., 17, 57–95.

Carvalho, P. (1998). Contribution to the lichen flora of Portugal. Lichens from Serra de S. Mamede Natural Park [Beitrag zur Flechtenflora von Portugal. Flechten aus dem Naturpark Serra de S. Mamede]. Sauteria, 9, 103–109.

Carvalho, P. (2001). Novos dados sobre a flora liquénica em Portugal. Actas do Colóquio de Fitotaxonomia. Instituto Superior de Agronomia, 22.

Carvalho, P. (2009). Estudo corológico do género *Collema* na Península Ibérica. Portugaliae Acta Biologica, 23, 67–146.

Carvalho, P. (2012). *Collema.* Flora Liquenológica Ibérica, 10. Sociedad Española de Liquenología.

Carvalho, P. & Jones, M. (1997). New and interesting lichens from Portugal. Cryptogamie, Bryologie et Lichenologie, 18(4), 291–294.

Carvalho, P., Figueira, R., Jones, M., Sérgio, C. & Sim-Sim, M. (2002). Biodiversidade da vegetação epifítica liquénica no litoral alentejano. Área de Sines. Portugaliae Acta Biologica, 20, 225–248.

Carvalho, P., Figueira, R., Jones, M., Sérgio, C., Sim-Sim, M. & Catarino, F. (2002). Dynamics of epiphytic lichen communities in an industrial area of Portugal. In S. (eds.) Llimona X/Lumbsch, HT/Ott (Ed.), Progress and Problems in Lichenology at the Turn of the Millenium (pp. 175–185). Bibliotheca Lichenologica, J. Cramer, Berlin, Stuttgart.

Carvalho, P., Figueira, R., Martins, A. & Calaim, J. (2007). A catalogue of Carlos Tavares Portuguese lichen collection housed at the Botanical Garden of the University of Lisbon Herbarium (LISU). Portugaliae Acta Biologica, 22, 5–145.

Catarino, F., Máguas, C., Sérgio, C., Branquinho, C. & Kratz, W. (1991). Lichens and bryophytes as bioindicators for air and water pollution in Portugal. In Proceedings of International Conference on Environmental Pollution, 1, 170–178. Intersciences Enterprises Ltd.

Clerc, P. (1984). *Usnea wirthii* - a new species of lichen from Europe and North Africa. Saussurea, 15, 33–36.

Clerc, P. (1991). *Usnea madeirensis* Mot. (ascomycète lichénisé): une espèce méconnue de l'Europe et de l'Amérique du Nord. Candollea, 46(2), 427–438.

Clerc, P. (1997). Notes on the genus *Usnea* Dill. Ex Adanson. The Lichenologist, 29(3), 209–215. https://doi.org/10.1006/lich.1996.0075

Clerc, P. (2011). Notes on the genus *Usnea* Adanson (lichenized Ascomycota). III. Bibliotheca Lichenologica, 106, 41–51.

Codogno, M. & Sancho, L. G. (1991). Distribution patterns of the lichen family *Umbilicariaceae* in the W Mediterranean Basin (Iberian Peninsula, S France and Italy). Botanika Chronika, 10, 901–910.

Colmeiro, M. (1867). Enumeración de las cryptógamas de Espana y Portugal. Revista de Los Progresos de Las Ciencias Exactas, 17–18, 63–164.

Colmeiro, M. (1889). Enumeración y revisión de las plantas de la Península Hispano-Lusitana é Islas Baleares, con la distribución geográfica de las especies, y sus nombres vulgares, tanto nacionales como provinciales, 5. Madrid.

Coppins, B. J. & James, P. W. (1989). The Identity of "*Lecidea cinnabarina*" in the British Isles. The Lichenologist, 21(3), 237–242.

Cordeiro, V. (1914). Lichens de Setúbal. Brotéria: Série Botanica, 12(1), 125–136.

Coutinho, A. X. P. C. (1916). Lichenum Lusitanorum Herbarii Universitatis Olisoponensis Catalogus. Lisboa. 122 pp.

Coutinho, A. X. P. C. (1917). Catalogui Lichenum Lusitanorum Herbarii - Suplementum Primum. Lisboa. 40 pp.

Crespo, A. (1979). Vegetación de líquenes epífitos mediterráneo ibero-atlánticos (*Pseudoparmelion soredianthis* al. nova). Documents Phytosociologiques, 4, 177–186.

Crespo, A., Blanco, O., Llimona, X., Zuzana Ferencová & Hawksworth, D. L. (2004). C*oscinocladium*, an overlooked endemic and monotypic mediterranean lichen genus of *Physciaceae*, Reinstated by Molecular Phylogenetic Analysis. Taxon, 53(2), 405–414. JSTOR. https://doi.org/10.2307/4135618

Crespo, A., Divakar, P. K., Argüello, A., Gasca, C. & Hawksworth, D. L. (2004). Molecular studies on *Punctelia* species of the Iberian Peninsula, with an emphasis on specimens newly colonizing Madrid. The Lichenologist, 36(5), 299–308. https://doi.org/10.1017/S0024282904014434

Crespo, A., Lumbsch, H. T., Mattsson, J.-E., Blanco, O., Divakar, P. K., Articus, K., Wiklund, E., Bawingan, P. A. & Wedin, M. (2007). Testing morphology-based hypotheses of phylogenetic relationships in *Parmeliaceae* (Ascomycota) using three ribosomal markers

and the nuclear RPB1 gene. Molecular Phylogenetics and Evolution, 44(2), 812–824. https://doi.org/10.1016/j.ympev.2006.11.029

Culberson, W. L. (1969). The behavior of the species of the *Ramalina siliquosa* group in Portugal. Österreichische Botanische Zeitschrift, 116(1–5), 85–94. https://doi.org/10.1007/BF01379613

Czeczuga, B., Etayo, J., Giralt, M., Casares, M., Lumbsch, H. T. & Salema, R. (1996). Carotenoids in the thalli of lichen species on the Iberian Peninsula. Feddes Repertorium, 107(1–2), 89–97. https://doi.org/10.1002/fedr.19961070112

dal Grande, F., Beck, A., Cornejo, C., Singh, G., Cheenacharoen, S., Nelsen, M. P. & Scheidegger, C. (2014). Molecular phylogeny and symbiotic selectivity of the green algal genus *Dictyochloropsis* s.l. (*Trebouxiophyceae*): a polyphyletic and widespread group forming photobiont-mediated guilds in the lichen family *Lobariaceae*. New Phytologist, 202(2), 455–470. https://doi.org/10.1111/nph.12678

Daveau, J. (1883). Catalogue de plantes qui croissent aux îles Berlengas et Farilhão Grande. Boletim da Sociedade Broteriana, 2, 17–31.

Degelius, G. (1954). The lichen genus *Collema* in Europe: morphology, taxonomy, ecology. Symbolae Botanicae Upsaliensis, 13, 1–499. https://api.semanticscholar.org/CorpusID:82610774

Dickhäuser, A., Lumbsch, H. T. & Feige, G. B. (1995). A synopsis of the *Lecanora subcarnea* group. Mycotaxon, 56, 303–323. https://biostor.org/reference/297486

Diederich, P. & Christiansen, M. S. (1994). *Biatoropsis usnearum* Räsänen, and other heterobasidiomycetes on *Usnea*. The Lichenologist, 26(1), 47–66. https://doi.org/10.1006/lich.1994.1004

Divakar, P. K., Blanco, O., Hawksworth, D. L. & Crespo, A. (2005). Molecular phylogenetic studies on the *Parmotrema reticulatum* (syn. *Rimelia reticulata*) complex, including the confirmation of *P. pseudoreticulatum* as a distinct species. The Lichenologist, 37(1), 55–65. Cambridge Core. https://doi.org/10.1017/S0024282904014586

Divakar, P. K., Figueras, G., Hladun, N. L. & Crespo, A. (2010). Molecular phylogenetic studies reveal an undescribed species within the North American concept of *Melanelixia glabra* (*Parmeliaceae*). Fungal Diversity, 42(1), 47–55. https://doi.org/10.1007/s13225-010-0027-3

Divakar, P., Molina, C., Lumbsch, T. & Crespo, A. (2005). *Parmelia barrenoae*, a new lichen species related to *Parmelia sulcata* (*Parmeliaceae*) based on molecular and morphological data. The Lichenologist, 37(1), 37–46.

Du Rietz, V. G. E. (1926). Flechtensystematische Studien VII. *Erioderma mollissimum* (Samp.) D.R. in Portugal, ein Repräsentant einer für Europa neuen Flechtengattung. Botaniska Notiser, 339–340.

Duvigneaud, P., Abbayes, H. des & Tavares, C. N. (1947). Un nouveau lichen méditerranéo-atlantique: *Cladonia mediterranea* P. Duvign. et des Abb., suivi de sa distribution au Portugal. Revue Bryologique et Lichénologique, 16, 95–104.

Egea, J. M. (1989). Las comunidades liquenicas saxicolas, ombrofobas, litorales, del suroeste de Europa y norte de Africa (*Roccelletea phycopsis* classis prov.). Studia Geobotanica, 9, 73–152.

Egea, J. M. & Torrente, P. (1993). The lichen genus *Bactrospora*. The Lichenologist, 25(3), 211–255. https://doi.org/10.1006/lich.1993.1028

Egea, J. M, & Torrente, P. (1994). El género de hongos liquenizados *Lecanactis* (Ascomycotina). Bibliotheca Lichenologica, 54, 1–205.

Ertz, D. & Van Den Boom, P. P. G. (2020). *Lecanographa atlantica* (*Arthoniales*, *Lecanographaceae*), a widespread and conspicuous but still undescribed lichen-forming fungus. Phytotaxa, 472(2), 147–158. https://doi.org/10.11646/phytotaxa.472.2.5

Estácio da Veiga, S. P. M. (1869). Plantas da Serra de Monchique observadas em 1866. Jornal de Sciencias Mathematicas Physicas e Naturaes, 2(6), 120–130. https://www.biodiversitylibrary.org/bibliography/75942

Esteves, L., Alves, L. & Aires Barros, L. (1991). Saxicolous lichenic vegetation in monuments as bioindicators of pollution. Environmental P, 1, 162–169. LRMH. https://www.lrmh.fr/Default/doc/SYRACUSE/141014/saxicolous-lichenic-vegetation-in-monuments-as-bioindicators-of-pollution

Etayo, J. & López de Silanes, M.E. (2020). Hongos liquenícolas del norte de Portugal, especialmente del Parque Natural Montesinho. Nova Acta Científica Compostelana, 27, 35–50. https://revistas.usc.gal/index.php/nacc/article/view/6752

Figueira, R., Branquinho, C., Pacheco, A. M. G., Sousa, A. J. & Catarino, F. (1994). Desenvolvimento de biomonitores de salinidade atmosférica. Actas da 4ª Conferência Nacional Sobre a Qualidade Do Ambiente, II, M36–M48.

Figueira, R., Branquinho, C., Pacheco, A., Sousa, A. J. & Catarino, F. (1995). Salinidade atmosférica: primeiras etapas de um programa de biomonitorização. Técnica, 2/95, 27–34.

Figueira, R., Catarino, F., Pacheco, A. M. G. & Sousa, A. J. (1998). In situ studies on sea-salt uptake by epiphytic lichens. Sauteria, 9, 143–150.

Figueira, R., Pacheco, A., Sousa, A. J., Branquinho, C. & Catarino, F. (1996). Lichen-based saltfall biomonitoring: calibration and time-space trends. Actas da 5ª Conferência Nacional sobre a Qualidade do Ambiente, 1, 853–862.

Figueira, R., Pacheco, A., Sousa, A. J., Branquinho, C. & Catarino, F. (1998). Assessment of two epiphytic lichens as saltfall biomonitors: Calibration of transplants. Ecotoxicology and Environmental Restoration, 1(2), 61–69.

Figueira, R., Pacheco, A., Sousa, A., Branquinho, C. & Catarino, F. (1995). First studies in assessing cryptogamic epiphytes as biomonitors of sea-salt deposition density. In Power,

H, Moussiopoulos, N. & Brebbia C. A. (eds.), Air Pollution III, 2. Air Pollution Engineering and Management (pp. 431–438). Computational Mechanics Publications, Ashurst, UK.

Figueira, R., Sousa, A., Brown, D., Catarino, F. & Pacheco, A. (1999). Natural levels of saline elements in lichens: determination of cellular fractions and their importance as saline tracers. Lichenologist, 31(2), 183–196.

Figueira, R., Sousa, A., Pacheco, A. & Catarino, F. (1999). Variability of sea-salt deposition assessed by lichen monitoring in the south-west Portuguese coast. In Brebbia, C. A., Jacobson, M. & Power H. (eds.), Air Pollution VII (pp. 599–607). WIT Press, Boston.

Fonseca, A. J., Pina, F., Macedo, M. F., Leal, N., Romanowska-Deskins, A., Laiz, L., Gómez-Bolea, A. & Saiz-Jimenez, C. (2010). Anatase as an alternative application for preventing biodeterioration of mortars: Evaluation and comparison with other biocides. International Biodeterioration & Biodegradation, 64(5), 388–396. https://doi.org/10.1016/j.ibiod.2010.04.006

Fos, S. (1998). Líquenes epífitos de los alcornocales ibéricos. Correlaciones bioclimáticas, anatómicas y densimétricas con el corcho de reproducción. Guineana. Revista de Botánica, 4, 1–158. https://doi.org/10.1387/guineana.20449

Fos, S. & Clerc, P. (2000). The lichen genus *Usnea* on *Quercus suber* in Iberian cork-oak forests. The Lichenologist, 32(1), 67–88. https://doi.org/10.1006/lich.1999.0242

Fos, S., Atienza, V., Sanz, M., Calatayud, V. & Barreno, E. (1994). Macrolíquenes epifitos sobre *Quercus suber* L. en los alcornocales del centro y sur de Portugal. Studia Botanica, 13, 139–145.

Freitas, M. C., Catarino, F. M., Branquinho, C. & Máguas, C. (1993). Preparation of a lichen reference material. Journal of Radioanalytical and Nuclear Chemistry, 169(1), 47–55. https://doi.org/10.1007/BF02046782

Freitas, M., Reis, M., Alves, L. & Wolterbeek, H. (1999). Distribution in Portugal of some pollutants in the lichen *Parmelia sulcata*. Environmental Pollution, 106, 229–235.

Frisch, A. (1996). The lichen genus *Thelotrema* Ach. in Europe. In E. E. Farkas, R. Lücking, & V. Wirth (Eds.), Scripta Lichenologica: Festschrift in Honour of Hannes Hertel (pp. 111–130). J. Cramer.

Fryday, A. M. & van den Boom, P. P. G. (2019). *Lecidea phaeophysata*: a new saxicolous lichen species from western and southern Europe with a key to saxicolous lecideoid lichens present on Atlantic coasts. The Lichenologist, 51(3), 193–204. Cambridge Core. https://doi.org/10.1017/S0024282919000070

Gaio-Oliveira, G., Branquinho, C. & Máguas, C. (1998). Sources of variability in sampling lichens for biomonitoring purposes. Cuadernos de Investigación Biológicas, 20, 319–322.

Gaio-Oliveira, G., Dahlman, L., Palmqvist, K. & Máguas, C. (2005). Responses of the lichen *Xanthoria parietina* (L.) Th. Fr. to varying thallus nitrogen concentrations. The Lichenologist, 37(2), 171–179. Cambridge Core. https://doi.org/10.1017/S0024282904014598

Garcia, C., Carvalho, P., Sérgio, C., Sim-Sim, M. & Diogo, A. (2005). Epiphytic bryophytes and lichens in *Quercus rotundifolia* Lam. woodlands of Portugal and their value as ecological indicators (M. Marchetti, Ed.). European Forest Institute.

Gerlach, A., da Silveira, R. M., Rojas, C. & Clerc, P. (2020). Naming and describing the diversity in the *Usnea cornuta* aggregate (lichenized Ascomycota, *Parmeliaceae*) focusing on Brazilian specimens. Plant and Fungal Systematics, 65(2), 272–302. https://doi.org/10.35535/pfsyst-2020-0024

Giralt, M. (2000). The Identity of *Buellia ericina* (Nyl.) Jatta and its generic position. The Lichenologist, 32(4), 309–316. Cambridge Core. https://doi.org/10.1006/lich.2000.0277

Giralt, M. (2001). The lichen genera *Rinodina* and *Rinodinella* (lichenized Ascomycetes, *Physciaceae*) in the Iberian Peninsula. Schweizerbart Science Publishers. http://www.schweizerbart.de//publications/detail/isbn/9783443580582/Bibliotheca_Lichenologica_Band_79_Giralt

Giralt, M. (2010). *Physciaceae* I: *Endohyalina*, *Rinodina* y *Rinodinella.* Flora Liquenológica Ibérica, 5. Sociedad Española de Liquenología.

Giralt, M. & Barbero, M. (1995). The saxicolous species of the genus *Rinodina* in the Iberian Peninsula containing atranorin, pannarin or gyrophoric acid. Mycotaxon, 56, 45–80.

Giralt, M. & Clerc, P. (2011). *Tetramelas thiopolizus* comb. nov. With a key to all known species of *Tetramelas*. The Lichenologist, 43(5), 417–425. https://doi.org/10.1017/S0024282911000363

Giralt, M. & Llimona, X. (1997). The saxicolous species of the genera *Rinodina* and *Rinodinella* lacking spot test reactions in the Iberian Peninsula. Mycotaxon, 62, 175–224.

Giralt, M. & Llimona, X. (2000). Two new corticolous species of *Buellia* (*Physciaceae*) from the Iberian Peninsula. Mycotaxon, 75, 181–194.

Giralt, M. & Matzer, M. (1994). The corticolous species of the genus *Rinodina* with biatorine or lecideine apothecia in southern Europe and Macaronesia. The Lichenologist, 26, 14.

Giralt, M. & Mayrhofer, H. (1994). Four corticolous species of the genus *Rinodina* (lichenized Ascomycetes, *Physciaceae*) containing atranorin in southern Europe and adjacent regions. Nova Hedwigia, 59, 129–142.

Giralt, M. & Mayrhofer, H. (1994). Four corticolous species of the genus *Rinodina* (lichenized Ascomycetes, *Physciaceae*) with polyspored asci. Herzogia, 10, 29–37. https://doi.org/10.1127/herzogia/10/1994/29

Giralt, M. & Mayrhofer, H. (1995). Some corticolous and lignicolous species of the genus *Rinodina* (lichenized Ascomycetes, *Physciaceae*) lacking secondary lichen compounds and vegetative propagules in Southern Europe and adjacent regions. Bibliotheca Lichenologica, 57, 127–160.

Giralt, M. & Nordin, A. (2002). *Buellia triseptata* in the Iberian Peninsula. The Lichenologist, 34(2), 89–94.

Giralt, M. & van den Boom, P. P. G. (2013). *Buellia subericola*, a new species with triseptate ascospores from the Iberian Peninsula. The Lichenologist, 45(4), 477–482. Cambridge Core. https://doi.org/10.1017/S0024282913000182

Giralt, M., Barbero, M. & Elix, J. A. (2000). Notes on some corticolous and lignicolous *Buellia* species From the Iberian Peninsula. The Lichenologist, 32(2), 105–128. Cambridge Core. https://doi.org/10.1006/lich.1999.0251

Giralt, M., Barbero, M. & van den Boom, P. (1996). *Rinodina algarvensis*, a new saxicolous sorediate species from Portugal containing the stictic acid complex. The Lichenologist, 28(1), 1–8. https://doi.org/10.1017/S0024282996000035

Giralt, M., Etayo, J. & Gómez–Bolea, A. (2000). *Amandinea crassiuscula*, a new corticolous species from the Iberian Peninsula. The Lichenologist, 32(6), 521–529. Cambridge Core. https://doi.org/10.1006/lich.2000.0287

Giralt, M., Mayrhofer, H. & Obermayer, W. (1994). The species of the genus *Rinodina* (lichenized Ascomycetes, *Physciaceae*) containing pannarin in Eurasia with a special note on the taxonomy of *Rinodina granulans*. Mycotaxon, 50, 47–59.

Giralt, M., Mayrhofer, H. & Sheard, J. W. (1995). The corticolous and lignicolous sorediate, blastidiate and isidiate species of the genus *Rinodina* in southern Europe. The Lichenologist, 27(1), 3–24.

Giralt, M., Mayrhofer, H., van den Boom, P. P. G. & Elix, J. A. (2001). *Rinodina turfaceoides*, a new corticolous, blastidiate species from the Iberian Peninsula. The Lichenologist, 33(2), 97–102. Cambridge Core. https://doi.org/10.1006/lich.2000.0299

Giralt, M., Nordin, A. & Elix, J. A. (2010). A new chemotype of *Buellia triseptata* (*Physciaceae*). The Bryologist, 113(1), 72–76. https://doi.org/10.1639/0007-2745-113.1.72

Giralt, M., Paz-Bermúdez, G. & Elix, J. A. (2009). The saxicolous, xanthone-containing species of the genus *Buellia s.l.* (*Physciaceae*, Ascomycota) in the Iberian Peninsula. Nova Hedwigia, 89(3–4), 321–334. https://doi.org/10.1127/0029-5035/2009/0089-0321

Giralt, M., van den Boom, P. P. G. & Boqueras, M. (1996). Nuevas localidades para cinco especies del género *Rinodina* recientemente descritas o muy poco citadas. Cryptogamie, Bryologie et Lichénologie, 10, 5–9.

Giralt, M., van den Boom, P. P. G. & Elix, J. A. (2010). *Endohyalina*, the genus in the *Physciaceae* to accommodate the species of the *Rinodina ericina*-group. Mycological Progress, 9(1), 37–48. https://doi.org/10.1007/s11557-009-0616-2

Giralt, M., van den Boom, P. P. G. & Elix, J. A. (2011). *Amandinea myrticola*, a new corticolous species from Portugal. The Lichenologist, 43(3), 193–197. https://doi.org/10.1017/S0024282911000120

Grube, M. & Giralt, M. (1996). Studies on some species of *Arthothelium* occurring in the Western Mediterranean. The Lichenologist, 28(1), 15–36. https://doi.org/10.1006/lich.1996.0003

Guzow-Krzemińska, B., Sérusiaux, E., van den Boom, P. P. G., Brand, A. M., Launis, A., Łubek, A. & Kukwa, M. (2019). Understanding the evolution of phenotypical characters in the *Micarea prasina* group (*Pilocarpaceae*) and descriptions of six new species within the group. MycoKeys, 57, 1–30. https://doi.org/10.3897/mycokeys.57.33267

Hafellner, J. (1993). Die Gattung *Pyrrhospora* in Europa – eine erste Übersicht mit einem Bestimmungsschlüssel der Arten nebst Bemerkungen zu einigen außereuropäischen Taxa (lichenisierte Ascomycotina, Lecanorales). Herzogia, 9, 725–747.

Hafellner, J. (2009). *Phacothecium* resurrected and the new genus *Phacographa* (*Arthoniales*) proposed. Bibliotheca Lichenologica, 100, 85–121. http://www.schweizerbart.de//publications/detail/isbn/9783443580797/Bibliotheca_Lichenologica_Band_100

Hale, M. E. (1987). A monograph of the lichen genus *Parmelia* Acharius *sensu stricto* (Ascomycotina: *Parmeliaceae*). Smithsonian Institution Press. https://doi.org/10.5962/bhl.title.122568

Harmand, M. L. (1906). Notes relatives à la lichénographie du Portugal. Bulletin de La Societé Botanique de France, 53, 68–74.

Harmand, M. L. (1909). Notes relatives à la lichénologie du Portugal. Suite. Bulletin de La Société Botanique de France, 56, 7–14, 27–34, 82–90, 124–131, 213–218. https://doi.org/10.1080/00378941.1909.10831394

Henriques, J. A. (1884). A vegetação da Serra do Gerez. Boletim Da Sociedade Broteriana, 3, 155–225.

Henriques, J. A. (1884). A vegetação espontânea do Bussaco. Boletim Da Sociedade Broteriana, 3, 109–123.

Henriques, J. A. (1880). Contributiones ad floram cryptogamicam lusitanicam. Enumeratio methodica Algarum, Lichenum et Fungorum herbarii praecipue Horti Regii bot. universitatis Conimbricensis. Typis Academicis.

Henriques, J. A. (1883). Expedição Scientifica à Serra da Estrella em 1881. Secção de Botânica, 27–29. Imprensa Nacional.

Henriques, J. A. (1890). Flora Lusitanica exsiccata, Cent. IX e X. Boletim Da Sociedade Broteriana, 8, 141.

Henriques, J. A. (1890). Lista geral das espécies distribuídas pela Sociedade Broteriana nos primeiros dez annos decorridos (1880-1889). Boletim Da Sociedade Broteriana, 8, 9.

Hertel, H. (1969). Die Flechtengattung *Trapelia* Choisy. Herzogia, 1(2), 111–130. https://doi.org/10.1127/herzogia/1/1969/111

Hertel, H. (1970). Parasitische lichenisierte Arten der Sammelgattung *Lecidea* in Europa. Herzogia, 1(4), 405–438. https://doi.org/10.1127/herzogia/1/1970/405

James, P. W. & White, F. J. (1987). Studies on the genus *Nephroma* I. The European and Macaronesian Species. The Lichenologist, 19(3), 215–215. https://doi.org/10.1017/S0024282987000239

Jansen, J. (1993). Korstmossen in de Serra da Estrela. Buxbaumiella, 31(1), 7–15.

Jones, M. P. (1980). Epiphytic macrolichens of the Algarve, Portugal. The Lichenologist, 12(3), 253–275.

Jones, M. P. (1983). Epiphytic macrolichens of the Sines area, Alentejo, Portugal. Revista de Biologia, 12, 313–325.

Jones, M. P. (1985). Epiphytic lichens of the Algarve, Portugal. Portugaliae Acta Biologica, Sér. B, 14, 199–212.

Jones, M. P. (1999). Notes on the distribution and composition of epiphytic lichen communities with *Nephroma laevigatum* Ach. in Portugal. Portugaliae Acta Biologica, Sér. B, Sistemática, Ecologia, Biogeografia e Paleontologia, 18(1–4), 51–120.

Jones, M. P. (2002). Some information on the distribution of lichens in Portugal. Portugaliae Acta Biologica, 20, 121–224.

Jones, M. P., Catarino, F. M., Sérgio, C. & Bento-Pereira, F. (1981). The Sines industrial complex monitoring programme: a preliminary report. Environmental Monitoring and Assessment, 1(2), 163–173. https://doi.org/10.1007/BF00395121

Jorge, A. R. (1918). Contribuições para o estudo da flora liquenológica portuguesa I.

Jorge, A. R. (1919). Contribuições para o estudo da flora liquenológica portuguesa II. Lisboa.

Jørgensen, P. M. (1994). Further notes on european taxa of the lichen genus *Leptogium*, with emphasis on the small species. The Lichenologist, 26(1), 1–29.

Jørgensen, P. M. (2000). *Moelleropsis nebulosa* ssp. *frullaniae*, a new lichen for Europe. Cryptogamie Mycologie, 21(1), 49–52. https://doi.org/10.1016/S0181-1584(00)00102-0

Jørgensen, P. M. (2003). Conspectus familiae *Pannariaceae* (Ascomycetes lichenosae). Ilicifolia, 4, 1–78.

Jørgensen, P. M. & James, P. W. (1983). Studies on some Leptogium species of Western Europe. The Lichenologist, 15(2), 109–125. https://doi.org/10.1017/S0024282983000183

Jørgensen, P. M. & Jones, M. P. (2012). *Leptogium resupinans* new to Europe from Portugal. Herzogia, 25(1), 101–102. https://doi.org/10.13158/heia.25.1.2010.101

Jurga Motiejūnaitė & Piotr Grochowski. (2014). Miscellaneous new records of lichens and lichenicolous fungi. Herzogia, 27(1), 193–198. https://doi.org/10.13158/heia.27.1.2014.193

Kärnefelt, I. (1990). Isidiate taxa in the *Teloschistales* and their ecological and evolutionary significance. The Lichenologist, 22(3), 307–320. Cambridge Core. https://doi.org/10.1017/S0024282990000342

Kilias, R. (1981). Revision of rock encrusting associations of the lichen family *Catillaria* Massal. in Europe. Herzogia, 5(3–4), 209–448. https://doi.org/10.1127/herzogia/5/1981/209

Klement, O. (1958). *Parmelia mougeotii* Schaer. im Naturschutzgebiet Romberg bei Lohr/M. und ihre Verbreitung in Europa. Nachrichten des Naturwissenschaftlichen Museums Aschaffenburg, 58, 39–47.

Kondratyuk, S. Y. & Galloway, D. J. (1995). Two new lichenicolous fungi from *Lobaria* and *Sticta* (*Stictaceae*). In F. J. A. Daniels, M. Schulz, & J. Peine (Eds.), Flechten Follmann: Contributions to lichenology in honour of Gerhard Follmann (pp. 255–261). Geobotanical and Phytotaxonomical Study Group, Botanical Institute, University of Cologne.

Kratz, W., Máguas, C., Branquinho, C., Catarino, F. & Sérgio, C. (1991). Der einsatz von epiphyten und aquatischen moosen für das schwermetallmonitoring in Portugal. VDI-Berichte, 901, 119–132.

Kurokawa, S. (1962). A monograph of the genus *Anaptychia*. Beih. Nova Hedwigia, 6, 1–115.

Laundon, J. R. (1981). The species of *Chrysothrix*. The Lichenologist, 13(2), 101–121. https://doi.org/10.1017/S0024282981000169

Laundon, J. R. (1989). The species of *Leproloma* - the name for the *Lepraria membranacea* group. The Lichenologist, 21(1), 1–22. Cambridge Core. https://doi.org/10.1017/S0024282989000034

Lázaro e Ibiza, B. (1898). Nota sobre algunos líquenes de España y Portugal. Actas de la Sociedad Española de Historia Natural, 180–186; 200–205.

Lepista, Z. & Aptroot, A. (2016). Seven species of *Graphis* from Portugal reported new to Europe. The Lichenologist, 48(4), 259–267. Cambridge Core. https://doi.org/10.1017/S0024282916000153

Leuckert, C. & Kümmerling, H. (1991). Chemotaxonomische studien in der gattung *Leproloma* Nyl. ex Crombie (Lichenes). Nova Hedwigia, 52(1–2), 17–32.

Leuckert, Ch., Guse K. & Poelt J. (1969). Zur chemotaxonomie der *Pertusaria hymenea*-gruppe (Lichenes, *Pertusariaceae*). Herzogia, 1(2), 159–171. https://doi.org/10.1127/herzogia/1/1969/159

Llop, E. (2007). *Lecanorales*: *Bacidiaceae* I. *Bacidia* y *Bacidina*. Flora Liquenológica Ibérica, 3. Sociedad Española de Liquenología.

Llop, E. (2010). *Bacidia punica* (*Ramalinaceae*), a new corticolous species from the Mediterranean region. The Bryologist, 113(2), 365–370. https://doi.org/10.1639/0007-2745-113.2.365

Llop, E. & Hladun, N. (2000). Contribución al estudio del género *Bacidina* (Lichenes) en la Península Ibérica. Portugaliae Acta Biologica, 19, 267–275.

Llop, E. & Hladun, N. (2002). A key to Iberian species of the genus *Bacidia* with notes on some species. In S. (eds.) Llimona X/Lumbsch, HT/Ott (Ed.), Progress and Problems in Lichenology at the Turn of the Millenium (pp. 91–98). Bibliotheca Lichenologica, J. Cramer, Berlin, Stuttgart.

Löfgren, O. & Tibell, Leif. (1979). *Sphinctrina* in Europe. The Lichenologist, 11(2), 109–137.

Longán, A., Barbero, M. & Gómez-Bolea, A. (2000). Comparative studies on *Punctelia borreri*, *P. perreticulata*, and *P. subrudecta* (*Parmeliaceae*, lichenized Ascomycotina) from the Iberian Peninsula. Mycotaxon, 74(2), 367–378.

López de Silanes, M. E., Paz-Bermúdez, G., Carballal Durán, R. & Marques, J. (2012). The genus *Leptogium* (*Collemataceae*, Ascomycotina) in mainland Portugal. Sidowia, 64, 67–102.

López de Silanes, M. E. & Alvarez, J. (2003). The genus *Phaeographis* Mull. Arg. (*Graphidaceae*, Ascomycotina) in the Iberian Peninsula. Nova Hedwigia, 77(1–2), 147–160. https://doi.org/10.1127/0029-5035/2003/0077-0147

López de Silanes, M. E., Etayo, J. & Paz-Bermúdez, G. (2009). *Pronectria pilosa* (*Hypocreaceae*) sp. nov. and other lichenicolous fungi found on *Collemataceae* in the Iberian Peninsula. The Bryologist, 112(1), 101–108.

Lumbsch, H. T. (1988). The identity of *Diploschistes gypsaceus*. The Lichenologist, 20(1), 19–24. https://doi.org/10.1017/S0024282988000052

Lumbsch, H. T., Ahti, T., Altermann, S., Amo de Paz, G., Aptroot, A., Arup, U., Bárcenas Peña, A., Bawingan, P. A., Benatti, M. N., Betancourt, L. *et al.* (2011). One hundred new species of lichenized fungi: a signature of undiscovered global diversity. Phytotaxa, 18 (1), 1–127.

Machado, A., Šlejkovec, Z., Van Elteren, J. T., Freitas, M. C. & Baptista M. S. (2006). Arsenic speciation in transplanted Lichens and tree bark in the framework of a biomonitoring scenario. Journal of Atmospheric Chemistry, 53(3), 237–249. https://doi.org/10.1007/s10874-006-9013-2

Magnusson, A. H. (1929). A monograph of the genus *Acarospora*. Kungliga Svenska Vetenskapsakademiens Handlingar, 7(4), 1–400.

Magnusson, A. H. (1947). Studies in non-saxicolous species of *Rinodina* mainly from Europe and Siberia. Acta Horti Gothoburgensis, 17, 191–338.

Máguas, C., Kratz, W., Sérgio, C., Sim-Sim, M. & Catarino, F. (1990). The use of epiphytes for the monitoring of environmental heavy metal pollution in Portugal. Environmental Contamination – 4th International Conference, 254–256.

Mariz, J. (1889). Outra excursão botânica na mesma província. Boletim Da Sociedade Broteriana, 7, 35–85.

Marques, J. & Paz-Bermúdez, G. (2014). New and interesting lichen records for the Portuguese funga from the Upper Douro region (north-east Portugal). Austrian Journal of Mycology, 23, 37–53.

Marques, J., Hespanhol, H., Paz-Bermúdez, G. & Almeida, R. (2014). Choosing between sides in the battle for pioneer colonization of schist in the Côa Valley Archaeological Park: a community ecology perspective. Journal of Archaeological Science, 45, 196–206.

Marques, J., Schultz, M. & Paz-Bermúdez, G. (2013). A *Peltula* Nyl. diversity hotspot in north-east Portugal, with one species new to science and three species new to mainland Europe. The Lichenologist, 45(4), 483–496. https://doi.org/10.1017/S0024282913000261

Martínez, I. (2002). Lichenicolous fungi from the Iberian Peninsula and the Macaronesian area. Nova Hedwigia, 74(1–2), 51–67. https://doi.org/10.1127/0029-5035/2002/0074-0051

Martínez, I. & Burgaz, A. R. (1997). Additions to the chemistry, ecology and distribution of *Peltigera melanorrhiza* and *P. neopolydactyla*. Mycotaxon, 63, 389–394.

Martínez, I. & Burgaz, A. R. (2008). El género *Cladonia* en la península Ibérica. Supergrupo *Crustaceae*. Botanica Complutensis, 32, 21–36. https://revistas.ucm.es/index.php/BOCM/article/view/BOCM0808110021A

Martínez, I. & Hafellner, J. (1998). Lichens and lichenicolous fungi on *Peltigerales* in the Iberian Peninsula and the Canary Islands. Mycotaxon, 69, 271–310.

Martínez, I., (1998). Taxonomía del género *Peltigera* willd. (ascomycetes liquenizados) en la Península Ibérica y estudio de sus hongos liquenícolas. Teseis doctoral. Universidad Complutense de Madrid. https://produccioncientifica.ucm.es/documentos/5d1ffb222999521e412dcb8b

Martínez, I., Burgaz, A. R. & Vitikainen, O. (1997). Studies on the genus *Peltigera* in the Iberian Peninsula. II. Nova Hedwigia, 64(3–4), 367–391. https://doi.org/10.1127/nova.hedwigia/64/1997/367

Masson, D. (2005). Taxonomie, écologie et chorologie des espèces françaises des genera *Hypotrachyna* et *Parmelinopsis* (Ascomycota lichénisés, *Parmeliaceae*). Cryptogamie, Mycologie, 26(3), 205–263.

Masson, D. (2008). Découverte de cinq espèces rares de macrolichens dans les Pyrénées occidentales françaises: une présence relictuelle? Cryptogamie, Mycologie, 29(1), 35–61. https://sciencepress.mnhn.fr/sites/default/files/articles/pdf/cryptogamie-mycologie2008v29f1a5.pdf

Matos, P., Cardoso-Vilhena, J., Figueira, R. & Sousa, A. J. (2011). Effects of salinity stress on cellular location of elements and photosynthesis in *Ramalina canariensis* Steiner. The Lichenologist, 43(2), 155–164. Cambridge Core. https://doi.org/10.1017/S0024282910000757

Matos, P., Pinho, P., Aragón, G., Martínez, I., Nunes, A., Soares, A. M. V. M. & Branquinho, C. (2015). Lichen traits responding to aridity. Journal of Ecology, 103(2), 451–458. https://doi.org/10.1111/1365-2745.12364

Matzer, M. & Hafellner, J. (1990). Eine Revision der lichenicolen Arten der Sammelgattung *Rosellinia* (Ascomycetes). Bibliotheca Lichenologica, 37, 1–138.

Matzer, M., Mayrhofer, H., Sattler, J. & Clerc, P. (1994). *Rinodina canariensis* (lichenized Ascomycetes, *Physciaceae*), a new species parasitic on crustose lichens in Macaronesia and the Mediterranean region. Nordic Journal of Botany, 14(1), 105–111. https://doi.org/10.1111/j.1756-1051.1994.tb00576.x

Mayrhofer, H. (1979). Die saxicolen Arten der Flechtengattung *Rinodina* in Europa. Bibliotheca Lichenologica, 12, 1–186.

Mayrhofer, H., Matzer, M., Sattler, J. & Egea, J. M. (1993). A revision of the Atlantic-Mediterranean *Rinodina beccariana* and related taxa (lichenized Ascomycetes, *Physciaceae*). Nova Hedwigia, 57(3–4), 281–304.

Mayrhofer, H., Scheidegger, C. & Sheard, J. W. (1992). On the taxonomy of five saxicolous species of the genus *Rinodina* (lichenized Ascomycetes). Nordic Journal of Botany, 12(4), 451–459. https://doi.org/10.1111/j.1756-1051.1992.tb01326.x

Mayrhofer, H., Sheard, J. W., Grassler, M. C. & Elix, J. A. (2001). *Rinodina intermedia* (*Physciaceae*, Lichenized Ascomycetes): A well-characterized species with submuriform ascospores. The Bryologist, 104(3), 456–463. JSTOR. http://www.jstor.org/stable/3244779

McCune, B. (2002). *Hypogymnia bryophila*, a new sorediate lichen species from Portugal. The Bryologist, 105(3), 470–472. JSTOR. http://www.jstor.org/stable/3244701

Moberg, R. (1989). Studies on *Physcia* - III Three *Physcia*-species new to Europe and the Azores. Herzogia, 8(1–2), 249–253. https://doi.org/10.1127/herzogia/8/1989/249

Muñiz, D. & Hladún, N. (2011). *Calicioides*. Flora Liquenológica Ibérica, 7. Sociedad Española de Liquenología.

Munzi, S., Correia, O., Silva, P., Lopes, N., Freitas, C., Branquinho, C. & Pinho, P. (2014). Lichens as ecological indicators in urban areas: beyond the effects of pollutants. Journal of Applied Ecology, 51(6), 1750–1757. https://doi.org/10.1111/1365-2664.12304

Munzi, S., Varela, Z. & Paoli, L. (2019). Is the length of the drying period critical for photosynthesis reactivation in lichen and moss components of biological soil crusts? Journal of Arid Environments, 166, 86–90. https://doi.org/10.1016/j.jaridenv.2019.04.019

Navarro-Rosinés, P. & Roux, C. (1993). *Caloplaca tavaresiana* Nav.-Ros. et Roux sp. nov., espèce nouvelle de lichen du littoral de la région méditerranéenne. Nova Hedwigia, 57(1–2), 169–177.

Navarro-Rosinés, P. & Roux, C. (1995). *Caloplaca navasiana* Nav.-Ros. et Roux sp. nov., espèce nouvelle de lichen du littoral Méditerranéen. Cryptogamie, Bryologie-Lichénologie, 16(2), 89–97.

Nylander, W. (1880). Addenda nova ad Lichenographiam Europaeam. Continuatio tertia et tricesima. Flora, 63, 10–15.

Nylander, W. (1881). Addenda nova ad Lichenographiam Europaeam. Continuatio quinta et tricesima. Flora, 64, 2–8.

Nylander, W. (1888). Lichens du nord du Portugal. Boletim Da Sociedade Broteriana, 6, 198–249.

Obermayer, W. (2019). Lichenotheca Graecensis, Fasc. 25 (Nos 481-500). Fritschiana, 93, 1–7.

Oksuz, D. P., Aguiar, C. A. S., Tápia, S., Llop, E., Lopes, P., Serrano, A. R. M., Leal, A. I., Correia, O., Matos, P., Rainho, A., Branquinho, C., Correia, R. A. & Palmeirim, J. M. (2020). The contribution of small shrubby patches to the functional diversity of wood-pastures. Acta Oecologica, 108, 103626. https://doi.org/10.1016/j.actao.2020.103626

Oliveira, G. G., Branquinho, C., Máguas, C. & Correia, O. (1999). Spatial impact of atmospheric dust from a cement mill in Serra da Arrábida using lichens as biomonitors. Revista de Biologia (Lisboa), 17, 33–42.

Oliveira, M. A., Llop, E., Andrade, C., Branquinho, C., Goble, R., Queiroz, S., Freitas, M. C. & Pinho, P. (2020). Estimating the age and mechanism of boulder transport related with extreme waves using lichenometry. Progress in Physical Geography: Earth and Environment, 44(6), 870–897. https://doi.org/10.1177/0309133320927629

Otálora, M., Aragón, G., Martínez, I. & Molina, M. C. (2004). A new corticolous species of *Leptogium* (*Collemataceae*) from Spain. The Lichenologist, 36(3–4), 197–202.

Paoli, L., Pinho, P., Branquinho, C., Loppi, S. & Munzi, S. (2017). The influence of growth form and substrate on lichen ecophysiological responses along an aridity gradient. Environmental Science and Pollution Research, 24(34), 26206–26212. https://doi.org/10.1007/s11356-017-9361-2

Paz-Bermúdez, G. (2003). The identity of some lichens described from Portugal by G. Sampaio and W. Nylander. Nova Hedwigia, 77(1–2), 115–123. https://doi.org/10.1127/0029-5035/2003/0077-0115

Paz-Bermúdez, G. (2007). The identity of two lichen species described from Portugal by J. Harmand. The Bryologist, 110(1), 119–122.

Paz-Bermúdez, G. & Carballal, R. (2005). The portuguese specimens of the family *Parmeliaceae* (lichenized ascomycetes) in the PO herbarium (Oporto, Portugal). Nova Hedwigia, 81(1–2), 205–228. https://doi.org/10.1127/0029-5035/2005/0081-0205

Paz-Bermúdez, G. & Carballal, R. (2008). Los géneros foliáceos de la familia *Physciaceae* (Lichenes, Ascomycota) en el herbario PO (Universidade de Porto, Portugal). Nova Acta Científica Compostelana. Bioloxía, 17, 31–38.

Paz-Bermúdez, G. & Elix, J. A. (2004). A new species of *Parmotrema* (Ascomycota: *Parmeliaceae*) from Portugal. Mycotaxon, 89(2), 505–508.

Paz-Bermúdez, G. & Elix, J. A. (2009). *Buellia carballaliana* (*Physciaceae*), a new lignicolous species from Portugal. The Bryologist, 112(4), 845–849.

Paz-Bermúdez, G. & Giralt, M. (2010). The Portuguese crustose specimens of the *Physciaceae*, *Caliciaceae* excluded, (lichenized ascomycetes) in the PO herbarium. Sidowia, 62(1), 105–136.

Paz-Bermúdez, G., Aguiar-Branco, H. & Folhadela, E. (2002). Typification of names of lichen taxa described by G. Sampaio and some others, deposited in Porto Herbarium (PO). Taxon, 51(4), 771–785. JSTOR. https://doi.org/10.2307/1555037

Paz-Bermúdez, G., Carballal, R. & López de Silanes, M. E. (2008). The genus *Fuscopannaria* (*Pannariaceae*, lichenized Ascomycota) in the Iberian Peninsula. Candollea, 63(2), 269–280.

Paz-Bermúdez, G., Carballal, R. & Valcárcel, C. P. (2006). *Caloplaca peregrina* (*Teloschistaceae*), a synonym of *C. pellodella*. The Bryologist, 109(2), 185–187.

Paz-Bermúdez, G., López de Silanes, M. E., Terrón, A., Arroyo, R., Atienza, V., Brime, S. F., Burgaz, A. R., Carvalho, P., Figueras, G., Llop, E., Marcos, B., Pino-Bodas, R., Prieto, M., Rico, V. J., Férnandez-Salegui, A. B. & Seriná, E. (2009). Lichens and lichenicolous fungi in the Montesinho Natural Park, the Serra da Nogueira and the Rio Sabor Valley (Portugal). Cryptogamie, Mycologie, 30(3), 279–303.

Pereira, M. J., Soares, A. & Branquinho, C. (1999). Stochastic simulation of fugitive dust emissions. In Geostatistics Wollongong 96 – Proceedings of the Fifth International Geostatistics Congress, Wollongong, Australia, September 1996 (pp. 1055–1065).

Pereira, M., Branquinho, C. & Soares, A. (1995). Modelling air pollution in a copper mine by using physical and biological monitoring. Proceedings of the 1995 3rd International Conference on Air Pollution, 45–97.

Pinho, P., Martins-Loução, M.-A., Máguas, C. & Branquinho, C. (2014). Calibrating total nitrogen concentration in lichens with emissions of reduced nitrogen at the regional scale. In M. A. Sutton, K. E. Mason, L. J. Sheppard, H. Sverdrup, R. Haeuber, & W. K. Hicks (Eds.), Nitrogen Deposition, Critical Loads and Biodiversity (pp. 217–227). Springer Netherlands. https://doi.org/10.1007/978-94-007-7939-6_24

Poelt, J. (1983). *Bryonora*, eine neue Gattung der *Lecanoraceae*. Nova Hedwigia, 38, 73–111.

Poelt, J. & Kalb, K. (1985). Die Flechte *Caloplaca congrediens* und ihre Verwandten: Taxonomie, Biologie und VerbreitungHerrn Prof. Dr. H. Meusel zum 75. Geburtstag gewidmet. Flora, 176(1–2), 129–140. https://doi.org/10.1016/S0367-2530(17)30108-1

Poelt, J. & Leuckert, C. (1991). Der Formenkreis von *Protoparmelia atriseda* (Lichenes, *Lecanoraceae*) in Europa. Nova Hedwigia, 52, 39–64.

Poelt, J. & Petutsching W. (1992). *Xanthoria candelaria* und ähnliche Arten in Europa. Herzogia, 9(1–2), 103–114. https://doi.org/10.1127/herzogia/9/1992/103

Poelt, J. & Tavares, C. N. (1969). *Xanthoria resendei*, eine neue Laubflechte der südwesteuropäischen Küsten. Portugaliae Acta Biologica, 9(3–4), 300–308.

Prieto, M., Aragón, G. & Martínez, I. (2010). The genus *Catapyrenium* s. lat. (*Verrucariaceae*) in the Iberian Peninsula and the Balearic Islands. The Lichenologist, 42(6), 637–684. Cambridge Core. https://doi.org/10.1017/S0024282910000319

Printzen, C. (1999). *Japewiella* gen. nov., a new lichen genus and a new species from Mexico. The Bryologist, 102(4), 714–719. JSTOR. https://doi.org/10.2307/3244257

Printzen, C., Spribille, T. & Tønsberg, T. (2008). *Myochroidea*, a new genus of corticolous, crustose lichens to accommodate the *Lecidea leprosula* group. The Lichenologist, 40(3), 195–207. Cambridge Core. https://doi.org/10.1017/S0024282908007639

Rico, V. J. & Barrasa, J. M. (2011). Basidiomycota liquenizados y liquenícolas. *Agaricales*: *Hygrophoraceae*. *Cantharellales*: *Clavulinaceae* e *Hydnaceae.* Flora Liquenológica Ibérica, 9. Sociedad Española de Liquenología.

Rico, V. J., Calatayud, A. & Giralt, M. (2006). The lichen genus *Dimelaena* (*Physciaceae*, *Lecanorales*) in the Iberian Peninsula. Cryptogamie, Mycologie, 27(2), 149–166.

Rico, V., Calatayud, V. & Giralt, M. (2003). *Buellia tesserata* and *Dimelaena radiata*, two closely related species. The Lichenologist, 35(2), 117–124.

Rico, V., van den Boom, P. P. G. & Barrasa, J. M. (2005). Morphology, chemistry and distribution of *Melanelia sorediella* (*Parmeliaceae*) and similar species in the Iberian Peninsula. The Lichenologist, 37(3), 199–215.

Rodrigues, S. A., Elix, J., Vingada, J. V. & Soares, A. M. V. M. (2007). The first records of *Hypotrachyna lividescens* and *H. pseudosinuosa* in the Iberian Peninsula. Cryptogamie, Mycologie, 28(2), 155–157.

Rodrigues, S. A., Terrón-Alfonso, A., Elix, J. A., Pérez-Ortega, S., Tønsberg, T., Fernández-Salegui, A. B. & Soares, A. M. V. M. (2011). *Lecanora sorediomarginata*, a new epiphytic lichen species discovered along the Portuguese coast. The Lichenologist, 43(2), 99–111. Cambridge Core. https://doi.org/10.1017/S002428291000071X

Rodrigues, S. A., Tønsberg, T., Terrón-Alfonso, A. & Soares, A. M. V. M. (2011). *Chrysothrix flavovirens*, *Lepraria elobata*, and *Ochrolechia arborea* new to Portugal. Mycotaxon, 115(1), 335–344. https://doi.org/10.5248/115.335

Roux, C. & Navarro-Rosinés, P. (1992). *Caloplaca egeana* Roux et Nav.-Ros. sp.nov. kaj *Caloplaca veneris* Roux et Nav.-Ros. sp.nov., du novaj likenspecioj de la mediteranea marbordo. Bulletin de La Société Linnéenne de Provence, 43, 97–103.

Roux, C., Navarro-Rosinés, P. & Giralt, M. (1992). Some interesting Mediterranean lichens and fungi from the Ports de Beseit range (Catalonia, Spain). Candollea, 47, 239–261.

Runemark, H. (1956). Studies in *Rhizocarpon*. II. Distribution and Ecology of the Yellow Species in Europe. Almqvist & Wiksell.

Sales, F. & Hedge, I. (2000). A floristic island in western Portugal. Flora Mediterranea, 10, 311–324.

Salisbury, G. (1971). *Thelotrema wightii* (T. Tayl.) Nyl. Portugaliae Acta Biologica, 11, 35–37.

Sampaio, G. (1916). Três líquenes novos. Revista dos estudos da Universidade do Porto, 2, 37.

Sampaio, G. (1917). Espécies novas de líquenes. Annaes da Academia Polytechnica do Porto, 12(1), 47–50. http://www.fc.up.pt/fa/index.php?p=nav&f=books.0153.W_0153_000025#faimg

Sampaio, G. (1917). Líquenes novos para a flora portuguesa. 2a série. Brotéria: Sér. Bot., 15(1), 12–29.

Sampaio, G. (1917). Líquenes novos para a flora portuguesa. 3a série. Brotéria: Sér. Bot., 15(3), 128–145.

Sampaio, G. (1918). Contribuições para o estudo dos líquenes Portugueses. Annaes da Academia Polytechnica do Porto, 13(1), 24–38. http://www.fc.up.pt/fa/index.php?p=nav&f=books.0154.W_0154_000015#faimg

Sampaio, G. (1918). *Psorotichia henriquesi* (n. sp.). O Instituto, Revista Scientifica e Literária, 65(5), 245.

Sampaio, G. (1920). Líquenes das regiões de Paredes de Coura. Boletim da Sociedade Broteriana, 28, 91–94.

Sampaio, G. (1920). Líquenes inéditos. Gabinete de Botânica da Faculdade de Sciências.

Sampaio, G. (1921). Novas contribuições para o estudo dos líquenes portugueses. Brotéria: Sér. Bot., 19(1), 12–35.

Sampaio, G. (1922). Materiais para a liquenologia portuguesa. Brotéria: Sér. Bot., 20, 147–163.

Sampaio, G. (1923). *Carlosia* Samp. novo género de líquenes. A Águia, 23(3), 192.

Sampaio, G. (1924). Novos materiais para a liquenologia Portuguesa. Boletim Da Sociedade Broteriana, 2.

Sancho, L. (1982). Nuevos datos sobre las Umbilicariáceas (Líquenes) Ibéricas. Collectanea Botanica, 13(1), 339–349.

Sancho, L. G. & Crespo, A. (1989). *Lasallia hispanica* and related species. The Lichenologist, 21(1), 45–58. Cambridge Core. https://doi.org/10.1017/S002428298900006X

Sanders, W. B. & Llop, E. (2020). *Gyalectidium minus* Sérus., new to oceanic western Europe in the first reported foliicolous lichen community of continental Portugal. The Lichenologist, 52(3), 247–249. Cambridge Core. https://doi.org/10.1017/S0024282920000080

Santesson, R. (1939). Amphibious pyrenolichens I. Arkiv För Botanik, 29A(10), 1–67.

Santos Silva, C. (1994). O género *Caloplaca* Th. Fr. em Portugal continental. Espécies novas ou pouco conhecidas. Studia Botanica, 13, 109–114.

Sarrión, F., Aragón, G. & Burgaz, A. (1999). Studies on mazaediate lichens and calicioid fungi of the Iberian Peninsula. Mycotaxon, 71, 169–198.

Scheidegger, C. & Ruef, B. (1988). Die xanthonhaltigen, gesteinsbewohnenden Sippen der Flechtengattung, *Buellia* De Not (*Physciaceae*, *Lecanorales*) in Europa. Nova Hedwigia, 47(3–4), 433–468.

Scheidegger, C. (1993). A revision of european saxicolous species of the genus *Buellia* De Not. and formerly included genera. The Lichenologist, 25(4), 315–364.

Sérgio, C. & Bento-Pereira, F. (1981). Líquenes e briófitos como bioindicadores da poluição atmosférica. I. Utilização de uma escala qualitativa para Lisboa. Boletim da Sociedade Broteriana (2a Série), LIV, 291–303.

Sérgio, C. & Sim-Sim, M. (1985). Estudo da poluição atmosférica no estuário do Tejo. A vegetação epifítica como bioindicadora. Portugaliae Acta Biologica (B), 14, 213–244.

Sérgio, C., Carvalho, P., Garcia, C. A., Almeida, E., Novais, V., Sim-Sim, M., Jordão, H. & Sousa, A. J. (2016). Floristic changes of epiphytic flora in the Metropolitan Lisbon area between 1980–1981 and 2010–2011 related to urban air quality. Ecological Indicators, 67, 839–852. https://doi.org/10.1016/j.ecolind.2016.03.022

Sérusiaux, E. (1989). Liste rouge des macrolichens dans la communauté européenne. Centre de recherche sur les Lichens, Département de Botanique.

Sérusiaux, E., Berger, F., Brand, M. & van den Boom, P. (2007). The lichen genus *Porina* in Macaronesia, with descriptions of two new species. Lichenologist, 39(1), 15–33.

Sim-Sim, M., Carvalho, P. & Sérgio, C. (2000). Cryptogamic epiphytes as indicators of air quality around an industrial complex in the Tagus valley, Portugal. Factor analysis and environmental variables. Cryptogamie Bryologie, 21(2), 153–170. https://doi.org/10.1016/S1290-0796(00)00110-3

Sim-Sim, M., Carvalho, P., Sérgio, C., Garcia, C. & Rego, F. (2004). Recolonisation and changes in bryophyte and lichen biodiversity in burned areas from the Serra da Estrela (Portugal). Cryptogamie, Bryologie, 25(4), 367–384.

Sipman, H. & Aptroot, A. (2020). *Ikaeria serusiauxii*, a new *Caloplaca*-like lichen from Macaronesia and mainland Portugal, with a lichen checklist for Porto Santo. Plant and Fungal Systematics, 65(1), 120–130. https://doi.org/10.35535/pfsyst-2020-0006

Sparrius, L. B., James, P. W. & Allen, M. A. (2005). The sorediate variety *of Sclerophytomyces circumscriptus*. The Lichenologist, 37(4), 285–289. Cambridge Core. https://doi.org/10.1017/S0024282905015082

Spjut, R., Simon, A., Guissard, M., Magain, N. & Sérusiaux, E. (2020). The fruticose genera in the *Ramalinaceae* (Ascomycota, *Lecanoromycetes*): their diversity and evolutionary history. MycoKeys, 73, 1–68. https://doi.org/10.3897/mycokeys.73.47287

Tavares, C. N. (1942). Alguns líquenes interessantes da região de Sintra. Boletim da Sociedade Broteriana, 16(2), 2.

Tavares, C. N. (1942). Notas liquenológicas III. Espécies novas ou interessantes para a flora liquenológica de Portugal. Brotéria: Ciências Naturais, 11(1), 42–48.

Tavares, C. N. (1944). Notes lichénologiques IV. Lichens nouveaux ou intéressants pour le Portugal. Boletim da Sociedade Broteriana, 19, 163–179.

Tavares, C. N. (1945a). Líquenes da Serra da Estrela (contribuição para o seu estudo). Brotéria: Ciências Naturais, 14(1/2), 14–25, 49–61.

Tavares, C. N. (1945b). Contribuição para o estudo das Parmeliáceas portuguesas. Portugaliae Acta Biologica, 1(1/2), 1–210.

Tavares, C. N. (1945c). Sobre algumas espécies do elemento oceânico em Portugal. Boletim da Sociedade Broteriana, 19, 493–502.

Tavares, C. N. (1947). Notes lichénologiques V. Portugaliae Acta Biologica, 2, 160–174.

Tavares, C. N. (1949). Notes lichénologiques VII. Portugaliae Acta Biologica, 154–164.

Tavares, C. N. (1950). Líquenes da Serra do Gerês. Portugaliae Acta Biologica, Sér. B, 3(1–2), 1–189.

Tavares, C. N. (1965). The genus *Pannaria* in Portugal. Portugaliae Acta Biologica, 8(1–4), 1–16.

Tavares, C. N. (1939). Notes lichénologiques I. Boletim da Sociedade Portuguesa de Ciências Naturais, 13, 53–60.

Tavares, C. N. (1941). Notes lichénologiques II. Boletim da Sociedade Portuguesa de Ciências Naturais, 13, 157–167.

Tavares, C. N. (1944). Sobre algumas espécies dos géneros *Cladonia* e *Physcia*. Boletim da Sociedade Broteriana, 2a Série, 19, 191–199.

Tavares, C. N. (1947). Notes lichénologiques VI. Brotéria: Ciências Naturais, 16, 145–157.

Tavares, C. N. (1950). Líquenes da Serra do Gerês. Catálogo. Agronomia Lusitana, 12(1), 123–163.

Tavares, C. N. (1951). Lichenes Lusitaniae selecti exsiccati, Fasciculus I (Nos. 1-25). Instituto Botanico Universitatis Olisiponensis.

Tavares, C. N. (1951). Novas espécies de líquenes para a flora portuguesa. Revista da Faculdade de Ciências de Lisboa, 2a Série, 1(2), 199–214.

Tavares, C. N. (1951). Sobre duas espécies do género *Toninia* Th. Fr. Revista da Faculdade de Ciências de Lisboa, 2a Série, 1(2), 189–194.

Tavares, C. N. (1952). Lichenes Lusitaniae selecti exsiccati, Fasciculus II (Nos. 26-50). Instituto Botanico Universitatis Olisiponensis.

Tavares, C. N. (1954). Notes lichénologiques VIII. Revista da Faculdade de Ciências de Lisboa, 2a Série, C, 3(2), 365–378.

Tavares, C. N. (1955). Lichenes Lusitaniae selecti exsiccati, Fasciculus III (Nos. 51-75). Instituto Botanico Universitatis Olisiponensis.

Tavares, C. N. (1956). Lichenes Lusitaniae selecti exsiccati, Fasciculus IV (Nos. 76-100). Instituto Botanico Universitatis Olisiponensis.

Tavares, C. N. (1956). Notes lichénologiques IX. Revista da Faculdade de Ciências de Lisboa, 2a Série, C, 5(1), 123–134.

Tavares, C. N. (1957). "*Teloschistes bioreti* Des Abb." and the variation in *Anaptychia intricata* (Desf.) Mass. Portugaliae Acta Biologica, Série B, 6(1), 44–52.

Tavares, C. N. (1958). Lichenes Lusitaniae selecti exsiccati, Fasciculus V (Nos. 101-125). Instituto Botanico Universitatis Olisiponensis.

Tavares, C. N. (1958). On the taxonomy of some *Roccella* species of the "*canariensis*" group. Revista da Faculdade de Ciências de Lisboa, 2a Série, C, 6(2), 125–144.

Tavares, C. N. (1960). About the occurrence of **Coccocarpia parmelioides** (Hook.) Trevis. s. l. in Portugal and Morocco. Portugaliae Acta Biologica, 6(1), 1–10.

Tavares, C. N. (1961). Lichenes Lusitaniae selecti exsiccati, Fasciculus VI (Nos. 126-150). Instituto Botanico Universitatis Olisiponensis.

Tavares, C. N. (1962). Lichenes Lusitaniae selecti exsiccati, Fasciculus VII (Nos. 151-175). Instituto Botanico Universitatis Olisiponensis.

Tavares, C. N. (1962). Novos dados sobre o género *Parmelia* em Portugal. Adições e correcções. Broteria, Série de Ciências Naturais, 31(1), 1–8.

Tavares, C. N. (1964). Lichenes Lusitaniae selecti exsiccati, Fasciculus VIII (Nos. 176-200). Instituto Botanico Universitatis Olisiponensis.

Tavares, C. N. (1965). Lichenes Lusitaniae selecti exsiccati, Fasciculus IX (Nos. 201-225). Instituto Botanico Universitatis Olisiponensis.

Tavares, C. N. (1968). Lichenes Lusitaniae selecti exsiccati, Fasciculus X (Nos. 226-250). Instituto Botanico Universitatis Olisiponensis.

Tehler, A. (1983). The genera *Dirina* and *Roccellina* (*Roccellaceae*). Nordic Journal of Botany, 3(5), 628–628. https://doi.org/10.1111/j.1756-1051.1983.tb01473.x

Tehler, A. (1988). *Dirina massiliensis* f. *aponina* (Massal.) Tehler, a pycnidial anamorph in the lichen genus *Dirina* (*Roccellaceae*). Lichenologist, 20(4), 398–399.

Tehler, A., Dahlkild, Å., Eldenäs, P. & Feige, G. B. (2004). The phylogeny and taxonomy of Macaronesian, European and Mediterranean *Roccella* (*Roccellaceae*, *Arthoniales*). Acta Universitatis Upsaliensis, Symbolae Botanicae Upsalienses, 34(1), 405–428.

Thor, G. (1985). A new species of *Lichenostigma*, A lichenicolous Ascomycete. The Lichenologist, 17(3), 269–272. https://doi.org/10.1017/S0024282985000342

Tibell, L. (1976). The genus *Thelomma*. Botaniska Notiser, 129, 221–249.

Tibell, L. (1999). Two new species of *Calicium* from Europe. Mycotaxon, 70, 431–443.

Timdal, E. (1991). A monograph of the genus *Toninia* (*Lecideaceae*, Ascomycetes). Opera Botanica, 110, 137 pp.

Torrente, P. & Egea, J. (1991). *Llimonaea*, a new genus of lichenized fungi in the order *Opegraphales* (Ascomycotina). Nova Hedwigia, 52(1–2), 239–245.

Torrente, P. & Egea, J. (1992). New species of *Opegrapha* from south-western Europe and northern Africa. Mycotaxon, 45, 83–92.

Torrente, P. & Egea, J. M. (1989). La familia *Opegraphaceae* en el area mediterránea de la Península Ibérica y Norte de Africa. Bibliotheca Lichenologica, 32, 1–282.

van den Boom, P. P. G. (2003). Contribution to the flora of Portugal, lichens and lichenicolous fungi III. Nova Hedwigia, 76(1–2), 157–171.

van den Boom, P. P. G. (2005). Contribution to the flora of Portugal, lichens and lichenicolous fungi IV. Cryptogamie, Mycologie, 26(1), 51–59.

van den Boom, P. P. G. (2010). *Waynea giraltiae*, a new lichen species from the Iberian Peninsula. The Lichenologist, 42(1), 29–33. Cambridge Core. https://doi.org/10.1017/S0024282909990326

van den Boom, P. P. G. (2016). Contribution to the flora of Portugal: lichens and lichenicolous fungi V. Österr. Z. Pilzk., 15, 11–19.

van den Boom, P. P. G. (2017). Lichens and lichenicolous fungi of Estremadura, Portugal, collected in 2015. Acta Botanica Hungarica, 59(3–4), 449–458. https://doi.org/10.1556/034.59.2017.3-4.11

van den Boom, P. P. G., Alonso, F. L. & Egea, J. M. (1996). *Lecania poeltii*, a new lichen species from Portugal and Northern Africa. The Lichenologist, 28(5), 395–399.

van den Boom, P. P. G., Aptroot, A. & Knaap, W. O. van der. (1990). New and interesting lichen records from Portugal. Nova Hedwigia, 50(3–4), 463–472.

van den Boom, P. P. G. & Brand, A. M. (2007). *Llimonaea sorediata*, a new lichen (Ascomycota), widely distributed in western Europe. The Lichenologist, 39(4), 309–309. https://doi.org/10.1017/S0024282907006871

van den Boom, P. P. G. & Brand, A. M. (2005). *Lecania fructigena* Zahlbr., a coastal saxicolous lichen, new for Europe, with notes on related species. The Lichenologist, 37(4), 277–283. Cambridge Core. https://doi.org/10.1017/S0024282905014842

van den Boom, P. P. G. & Brand, A. M. (2008). Some new *Lecanora* species from western and central Europe, belonging to the *L. saligna* group, with notes on related species. Lichenologist, 40(6), 465–497.

van den Boom, P. P. G., Brand, A. M., Coppins, B. J. & Sérusiaux, E. (2017). Two new species in the *Micarea prasina* group from Western Europe. The Lichenologist, 49(1), 13–25. Cambridge Core. https://doi.org/10.1017/S0024282916000633

van den Boom, P. P. G. & Coppins, B. J. (2001). *Micarea viridileprosa* sp. nov., an overlooked lichen species from Western Europe. The Lichenologist, 33(2), 87–91. https://doi.org/10.1006/lich.2000.0310

van den Boom, P. P. G. & Etayo, J. (1995). A new epiphytic species of the lichen genus *Caloplaca* from Southwestern Europe. Mycotaxon, 56, 125–134.

van den Boom, P. P. G. & Etayo, J. (2000). Contribution to the knowledge of lichenicolous fungi and lichens from Portugal and Spain. Österr. Z. Pilzk., 9, 151–162.

van den Boom, P. P. G & Etayo, J. (2001). Two new sorediate species of lichens in the *Catillariaceae* from the Iberian Peninsula. The Lichenologist, 33(2), 103–110. https://doi.org/10.1006/lich.2001.0307

van den Boom, P. P. G., Elix, J. A. & Giralt, M. (2021). Lichen diversity of crustose *Caliciaceae* and *Physciaceae* from Alentejo, the Azores and Madeira (Portugal) including the new *Amandinea madeirensis*. Herzogia, 33(2), 420–431. https://doi.org/10.13158/heia.33.2.2020.420

van den Boom, P. P. G. & Ertz, D. (2012). Lichens and lichenicolous fungi from El Hierro (Canary islands), a survey, including five new species. Cryptogamie, Mycologie, 33(1), 59–97. https://doi.org/10.7872/crym.v33.iss1.2012.059

van den Boom, P. P. G. & Jansen, J. (2002). Lichens in the upper belt of the Serra da Estrela (Portugal). Österr. Z. Pilzk., 11.

van den Boom, P. P. G. & Giralt, M. (1996). Contribution to the flora of Portugal, lichens and lichenicolous fungi I. Nova Hedwigia, 63, 145–172.

van den Boom, P. P. G. & Giralt, M. (1999). Contribution to the flora of Portugal, lichens and lichenicolous fungi II. Nova Hedwigia, 68(1–2), 183–196.

van den Boom, P. & Giralt, M. (2012). Checklist and three new species of lichens and lichenicolous fungi of the Algarve (Portugal). Sidowia, 64(2), 149–207.

van den Boom, P. P. G. & Llop, E. (2021). *Bacidina celtica* (*Ramalinaceae*), a new lichen species from western Europe. Sidowia, 74, 65–70.

van den Boom, P. P. G. & Rico, V. J. (2006). *Caloplaca squamuloisidiata*, a new lichen species from Portugal and Spain. The Lichenologist, 38(6), 529–535. https://doi.org/10.1017/S0024282906006153

van den Boom, P. P. G. & Vezda, A. (1995). A new species and a new variety of the lichen genus *Gyalidea* from western Europe. Mycotaxon, 54, 421–426.

Vandelli, D. (1788). Florae Lusitanicae et Brasiliensis specimen: et epistolae ab eruditis viris Carolo a Linné, Antonio de Haen ad Dominicum Vandelli scriptae. Ex typographia Academico-Regia, apud bibliopolam Antonium Barneoud. https://www.biodiversitylibrary.org/item/108418#page/8/mode/1up

Vandelli, D. (1789). Viridarium Grisley Lusitanicum, Linnaeanis nominibus illustratum, jussu Academiae in lucem editum. Ex Typographia Regalis Academiae Scientiarum Olisiponensis. http://bibdigital.rjb.csic.es/ing/Libro.php?Libro=4748

Vaz, A. S., Marques, J. & Honrado, J. P. (2014). Patterns of lichen diversity in coastal sand-dunes of northern Portugal. Botanica Complutensis, 38, 89–96.

Verseghy, K. (1962). Die Gattung *Ochrolechia*. Beihefte Zur Nova Hedwigia, 1, 1–146.

Virville, D. (1938). Les zones de lichens sur les côtes du Portugal. Boletim da Sociedade Broteriana, 13(2), 123–177.

Vitikainen, O. (1994). Taxonomic revision of *Peltigera* (lichenized Ascomycotina) in Europe. Acta Botanica Fennica, 152, 1–96.

Vondrák, J., Frolov, I., Košnar, J., Arup, U., Veselská, T., Halıcı, G., Malíček, J. & Søchting, U. (2020). Substrate switches, phenotypic innovations and allopatric speciation formed taxonomic diversity within the lichen genus *Blastenia*. Journal of Systematics and Evolution, 58(3), 295–330. https://doi.org/10.1111/jse.12503

Werner, R. G. (1996). *Wernerella* gen. nov. (*Dothideales*, Ascomycetes), un género para *incluir Leptosphaeria maheui*. Bulletin de La Société Linnéenne de Provence, 47, 167–177.
