# Supplementary material for: Checklist of Lichens and Lichenicolous Fungi from Mainland Portugal
Source: J Fungi (Basel). 2026 Jul 23;12(8):549. doi: 10.3390/jof12080549 (PMC13514346; doi:10.3390/jof12080549)
Supplement: Supplementary file 1 [file jof-12-00549-s001.zip › Table_S4.pdf]

Table S4: Taxa excluded from the Mainland Portugal after critical assessment of occurrences. Citations in the column "Reason" refer to Table S1.

Taxon	Reason	Occurrence References
Buellia geophila (Flörke ex Sommerf.) Lynge	Cited, as *Buellia triphragmia* (Nyl.) Arnol, by Sampaio in 1918 but according to Giralt (2010) it is *Buellia triseptata* A. Nordin	Sampaio, G. (1918). Contribuições para o estudo dos líquenes Portugueses. Annaes da Academia Polytechnica do Porto, 13(1), 24–38.
Cladonia hammeri Ahti	According to Burgaz et al. (2020) all the specimens cited as *Cladonia hammeri* correspond to *Cladonia humilis* (With.) J.R. Laundon.	Burgaz, A. R. & Ahti, T. (2009). *Cladoniaceae*. Flora Liquenológica Ibérica, 4. Sociedad Española de Liquenología.Paz-Bermúdez, G., López de Silanes, M. E., Terrón, A., Arroyo, R., Atienza, V., Brime, S. F., Burgaz, A. R., Carvalho, P., Figueras, G., Llop, E., Marcos, B., Pino-Bodas, R., Prieto, M., Rico, V. J., Férnandez-Salegui, A. B. & Seriná, E. (2009). Lichens and lichenicolous fungi in the Montesinho Natural Park, the Serra da Nogueira and the Rio Sabor Valley (Portugal). Cryptogamie, Mycologie, 30(3), 279–303.<br>van den Boom, P. & Giralt, M. (2012). Checklist and three new species of lichens and lichenicolous fungi of the Algarve (Portugal). Sidowia, 64(2), 149–207.
Allantoparmelia alpicola (Th. Fr.) Essl.	According to Paz-Bermudez & Carballal (2005) the specimens cited by Henriques (1918) and Sampaio (1917), as *Parmelia alpicola* Th. Fr., correspond to *Brodoa atrofusca* (Schaer.) Goward	Henriques, J. A. (1883). Expedição Scientifica à Serra da Estrella em 1881. Secção de Botânica, 27–29. Imprensa Nacional.<br>Sampaio, G. (1917). Líquenes novos para a flora portuguesa. 2a série. Brotéria: Sér. Bot., 15(1), 12–29.
Hypogymnia austerodes (Nyl.) Räsänen	According to C.N. Tavares (1945b) *Parmelia austerodes* Nyl. cited by Coutinho (1916) it is *Hypogymnia physodes* (L.) Nyl.	Coutinho, A. X. P. C. (1916). Lichenum Lusitanorum Herbarii Universitatis Olisoponensis Catalogus. Lisboa. 120 pp.
Parmotrema cetratum (Ach.) Hale	According to C.N. Tavares (1945b) all the specimens cited as *Parmelia cetrata* Ach. correspond to *Parmotrema reticulatum* (Taylor) M. Choisy	Jorge, A. R. (1919). Contribuições para o estudo da flora liquenológica portuguesa II. Lisboa.<br>Sampaio, G. (1920). Líquenes das regiões de Paredes de Coura. Boletim da Sociedade Broteriana, 28, 91–94.<br>Cordeiro, V. (1914). Lichens de Setúbal. Brotéria: Série Botanica, 12(1), 125–136.

Parmotrema latissimum (Fée) Hale	According to C.N. Tavares (1945b), *Parmelia latissima* Fée, cited by Coutinho, was misidentifed	Coutinho, A. X. P. C. (1916). Lichenum Lusitanorum Herbarii Universitatis Olisoponensis Catalogus. Lisboa. 120 pp.
Parmotrema saccatilobum (Taylor) Hale	According to C.N. Tavares (1945b), all the specimens cited as *Parmelia saccatiloba* Taylor in occurrence references were misidentified. According to Paz Bermudez & Elix (2004) and Paz-Bermúdez & Carballal (2005) the specimen cited by Nylander it is *Parmotrema sampaioi* Paz-Berm. & Elix	Harmand, M. L. (1909). Notes relatives à la Lichénologie du Portugal. Suite. Bulletin de La Société Botanique de France, 56, 7–14, 27–34, 82–90, 124–131, 213–218. https://doi.org/10.1080/00378941.1909.10831394<br>Colmeiro, M. (1889). Enumeración y revisión de las plantas de la Península Hispano-Lusitana é Islas Baleares, con la distribución geográfica de las especies, y sus nombres vulgares, tanto nacionales como provinciales, 5. Madrid.<br>Nylander. (1888). Lichens du nord du Portugal. Boletim da Sociedade Broteriana, 6, 198–249.
Parmotrema perforatum (Jacq.) A. Massal.	According to C.N. Tavares (1945b), all the specimens cited as *Parmelia perforata* (Jacq.) Ach. or *Parmelia perlata* (Huds.) Ach. var. *perforata* (Jacq.) Koltz in occurrences references correspond to *Parmotrema perlatum* (Huds.) M. Choisy	Nylander. (1888). Lichens du nord du Portugal. Boletim da Sociedade Broteriana, 6, 198–249.<br>Colmeiro, M. (1867). Enumeración de las cryptógamas de Espana y Portugal. Revista de Los Progresos de Las Ciencias Exactas, 17–18, 63–164.<br>Coutinho, A. X. P. C. (1916). Lichenum Lusitanorum Herbarii Universitatis Olisoponensis Catalogus. Lisboa. 120 pp..<br>Harmand, M. L. (1909). Notes relatives à la Lichénologie du Portugal. Suite. Bulletin de La Société Botanique de France, 56, 7–14, 27–34, 82–90, 124–131, 213–218. https://doi.org/10.1080/00378941.1909.10831394<br>van den Boom, P. & Giralt, M. (2012). Checklist and three new species of lichens and lichenicolous fungi of the Algarve (Portugal). Sidowia, 64(2), 149–207.
Usnea schadenbergiana Göpp. & Stein.	Erroneous citation of Jones (1980). Jones refered *Usnea hesperina* Motyka a synonym of *Usnea subgracilis* Vain.	van den Boom, P. & Giralt, M. (2012). Checklist and three new species of lichens and lichenicolous fungi of the Algarve (Portugal). Sidowia, 64(2), 149–207.
